# Tweedle proteins form extracellular two-dimensional structures defining body and cell shape in *Drosophila melanogaster*

**DOI:** 10.1098/rsob.200214

**Published:** 2020-12-09

**Authors:** Renata Zuber, Yiwen Wang, Nicole Gehring, Slawomir Bartoszewski, Bernard Moussian

**Affiliations:** 1 Applied Zoology, Technical University of Dresden, Zellescher Weg 20b, 01062 Dresden, Germany; 2 Interfaculty Institute for Cell Biology (Ifiz), University of Tübingen, Auf der Morgenstelle 15, 72076 Tübingen, Germany; 3 Department of Biochemistry and Cell Biology, Rzeszow University, ul. Zelwerowicza 4, 35-601 Rzeszów, Poland; 4 CNRS, Inserm, Institute of Biology Valrose, Université Côte d'Azur, Parc Valrose, 06108 Nice CEDEX 2, France

**Keywords:** *Drosophila*, ECM, cuticle, body shape, epidermis

## Abstract

Tissue function and shape rely on the organization of the extracellular matrix (ECM) produced by the respective cells. Our understanding of the underlying molecular mechanisms is limited. Here, we show that extracellular Tweedle (Twdl) proteins in the fruit fly *Drosophila melanogaster* form two adjacent two-dimensional sheets underneath the cuticle surface and above a distinct layer of dityrosinylated and probably elastic proteins enwrapping the whole body. Dominant mutations in *twdl* genes cause ectopic spherical aggregation of Twdl proteins that recruit dityrosinylated proteins at their periphery within lower cuticle regions. These aggregates perturb parallel ridges at the surface of epidermal cells that have been demonstrated to be crucial for body shaping. In one scenario, hence, this disorientation of epidermal ridges may explain the squatty phenotype of Twdl mutant larvae. In an alternative scenario, this phenotype may be due to the depletion of the dityrosinylated and elastic layer, and the consequent weakening of cuticle resistance against the internal hydrostatic pressure. According to Barlow's formula describing the distribution of internal pressure forces in pipes in dependence of pipe wall material properties, it follows that this reduction in turn causes lateral expansion at the expense of the antero-posterior elongation of the body.

## 1. Introduction

Extracellular matrices (ECM) contribute to the geometry and consistency of cells and tissues. Generally, the role of an ECM depends on its components and their interactions that ultimately define its organization. Cartilaginous tissues, for instance, consist of a random distribution of the extracellular polysaccharide hyaluronic acid and associated proteins, including collagen and aggrecan produced and secreted by embedded chondrocytes [1–4]. Tension applied on this kind of tissues in concert with swelling forces entail the arrangement of the components and, finally, the shape of the tissue.

A less well-studied ECM is the cuticle of insects that outlines the shape of the organism. In some body regions such as the head capsule of caterpillars or the protective elytra of adult beetles, the hardness of the exoskeleton is sufficient to sustain the required shape. In some other body regions such as the ventral abdomen of many adult insects or the larval body, the respective shape does not only depend on the exoskeleton but involves the inner hydrostatic pressure. Despite these differences the principal organization of the cuticle is well conserved in different body regions and among species. The prototype of the cuticle consists of three composite horizontal layers: the outermost envelope, the middle epicuticle and the inner procuticle [5,6]. Which factors account for the physical differences of the cuticle in different body regions is a matter of current research.

Like cartilaginous tissues, the procuticle is composed of an extracellular polysaccharide, namely chitin and associated proteins. In the last few years, data accumulated underlining that the proteins binding to chitin and their partners together specify the physical properties of the cuticle. In the elytral procuticle of the red flour beetle *Tribolium castaneum*, for instance, the chitin-binding proteins Cpr27 and Cpr18 associate with chitin and interact with the cuticle protein CP30 probably over covalent N-β-alanyl-dopamine (NBAD) bridges that are catalysed by the phenoloxidase TmLaccase2 [7,8]. This arrangement correlates with the stiffness of the elytral cuticle. In the fruit fly *Drosophila melanogaster*, proteins such as the chitin-binding and elastic protein Resilin in the contact region between the procuticle and the epicuticle are cross-linked to each other via dityrosine bonds [9,10]. The formation of this sub-layer involves the C-type lectin Schlaff (Slf) and a yet unknown peroxidase. The dityrosine sub-layer may be important for cuticle elasticity and thereby contributes to its shape.

In order to deepen our understanding of the mechanisms of cuticle ECM organization, we have studied the function of several members of a class of cuticle proteins named Tweedle (Twdl) in the fruit fly *D. melanogaster* [11]. Twdl proteins are characterized by an N-terminal signal peptide and a domain with four conserved blocks (DUF243), but do not display any homology to any other entry in protein databases. Deletions of stretches of amino acids of these domains provoke body shape changes in larvae and adult flies. In brief, we show that Twdl proteins localize to the epicuticle and assist the organization of the epicuticle–procuticle interface, which we hypothesize to be responsible for defining the body shape.

Here, we show that Twdl proteins localize to the epicuticle forming at least two two-dimensional sheets. In addition, we demonstrate that Twdl proteins with deletions in the DUF243 domain form ectopic aggregates within the procuticle. These aggregates recruit non-mutated Twdl proteins as well as proteins from the procuticle–epcuticle interface. We propose that this mis-organization entails changes in the physical properties of the cuticle that as a consequence dilates laterally, rather than longitudinally.

## 2. Results

### 2.1. Twdl proteins are expressed at different time points during development

**Figure 1.**
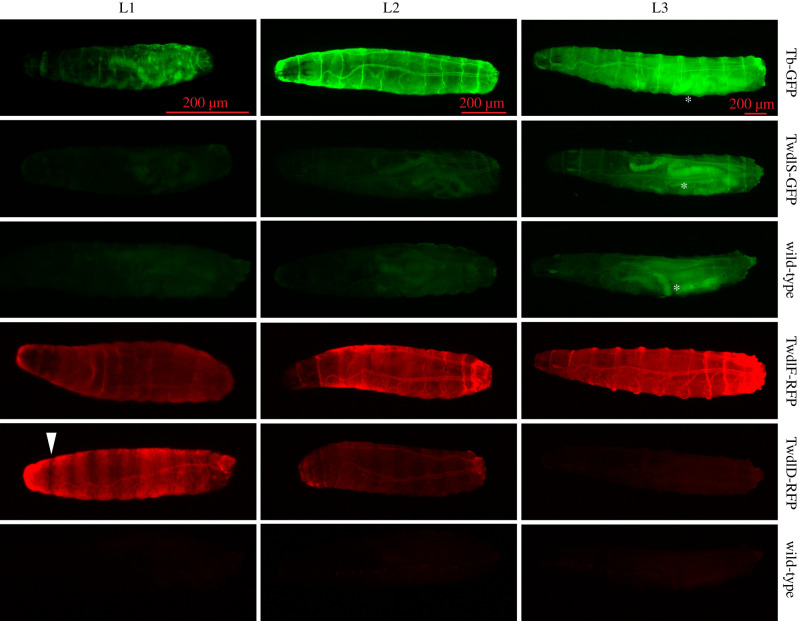
Expression profile of GFP- and dsRed-tagged Twdl proteins at three larval stages. All four Twdl proteins localize to the larval cuticle. Tb-GFP is weakly visible at first larval stage (L1) as lateral patches, while at second (L2) and third (L3) larval stages it is decently visible in the whole cuticle. The signal of TwdlS-GFP is very faint in the cuticle of L2 and weak in the whole cuticle of L3. TwdlF-RFP is observable in the entire cuticle at all larval stages. TwdlD-RFP shows strong striped signal in the cuticle of L1, weaker in L2 and very faint signal in the posterior cuticle of L3 larvae. Wild-type control larvae display a green auto-fluorescence in the green channel especially in the gut (*), while they hardly show any fluorescence in the red channel.

To study the cellular function of Twdl proteins, we first determined the expression pattern of transgenic flies expressing fluorescent-tagged versions of the candidates TwdlA-GFP (Tb-GFP), TwdlD-dsRed, TwdlF-dsRed and TwdlS-GFP under the control of their endogenous promoters (figure 1; see also ‘Material and methods’).

In the early larva (L1), the expression of Tb-GFP was patchy in the epidermis. During later larval stages (L2 and L3), Tb-GFP was found in the whole epidermis. The TwdlS-GFP signal was barely visible in the cuticle of L1 and L2 larvae and became more intense in the whole cuticle of L3 larvae. TwdlF-dsRed was localized in the whole cuticle at all three larval stages. TwdlD-dsRed was expressed in the epidermis during the first two larval stages, but was excluded from the segmental grooves. At the third larval stage, only a very faint TwdlD-dsRed signal at the posterior part was detected.

In summary, Twdl proteins are expressed at different time points during development in different, partially overlapping regions of the epidermis.

### 2.2. Twdl proteins mark the epicuticle

**Figure 2.**
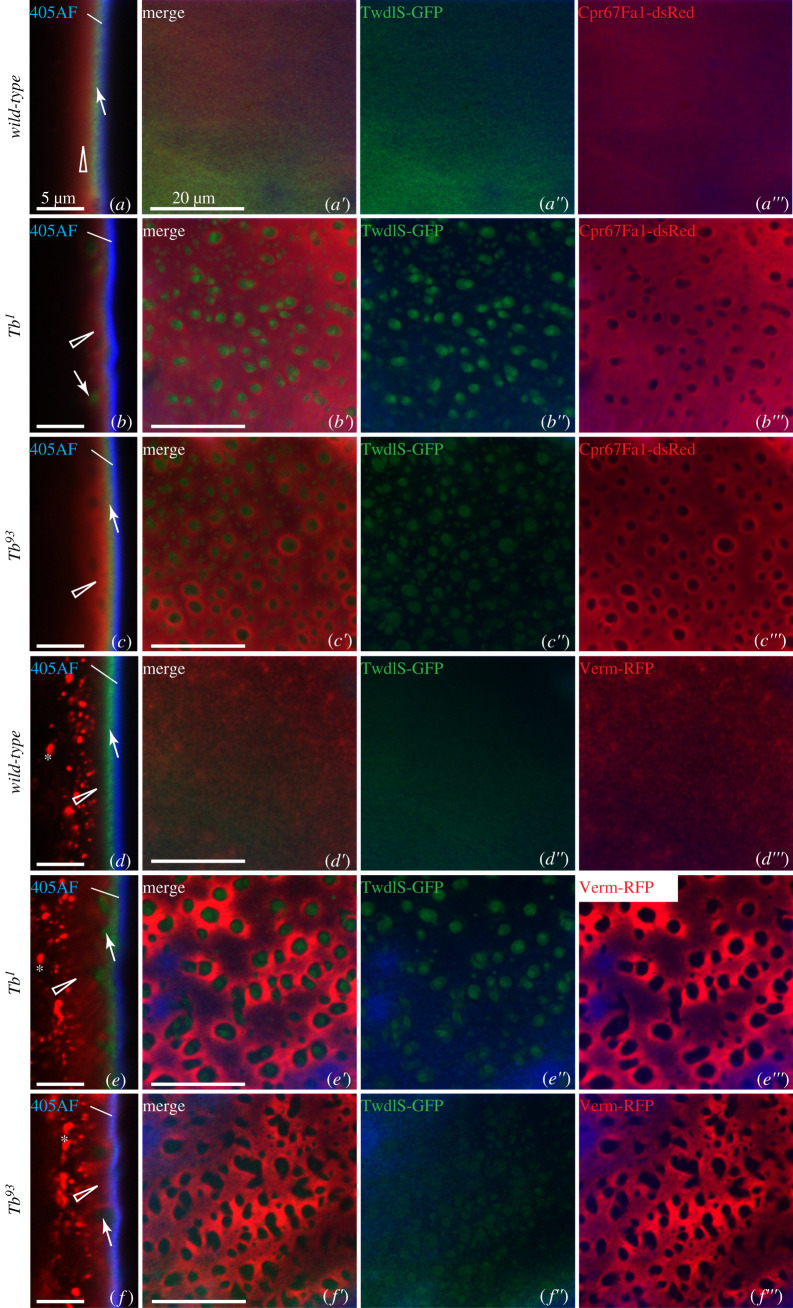
Localization of TwdlS-GFP, Cpr67Fa1-RFP and Verm-RFP in the cuticle of wild-type, Tb^1^ and Tb^93^ third instar larvae. (*a*) In an optical cross-section of the cuticle of wild-type larvae, TwdlS-GFP (green, arrow) is uniformly distributed forming a thin layer under the blue 405 nm-induced auto-fluorescent line that represents the envelope (405AF). dsRed-tagged chitin-binding Cpr67Fa1 (Cpr67Fa1-dsRed, red, triangle), expressed in the epidermis, is plainly distributed in the thick procuticle underneath the TwdlS-GFP layer. *a*′–*a*″′ show top views with separated channels. (*b*) In the cuticle of *Tb^1^* larvae (*b–b*″′) and *Tb^93^* larvae (*c–c*″′), TwdlS-GFP partially localizes to the upper epicuticle (arrow) and partially forms aggregates immersed in the procuticle marked by Cpr67Fa1-dsRed (triangle). The upper TwdlS-GFP layer does not overlap with the Cpr67Fa1-dsRed signal (*b*′–*b*″′,*c*′–*c*″′). (*d*) Ubiquitously expressed Vermiform-RFP (Verm-RFP, red, triangle) is plainly distributed in the entire procuticle below the TwdlS-GFP layer (arrow) and forms vesicle-like structures in the cells (*). *d*′–*d*″′ show top views with separated channels. In the cuticle of *Tb^1^* larvae (*e*–*e*″′) and *Tb^93^* larvae (*f–f*″′), TwdlS-GFP partially localizes to the upper epicuticle (arrow), and partially forms aggregates immersed in the procuticle (triangle). It does not overlap with the Verm-RFP signal (*e*′–*e*″′,*f*′–*f*″′).

**Figure 3.**
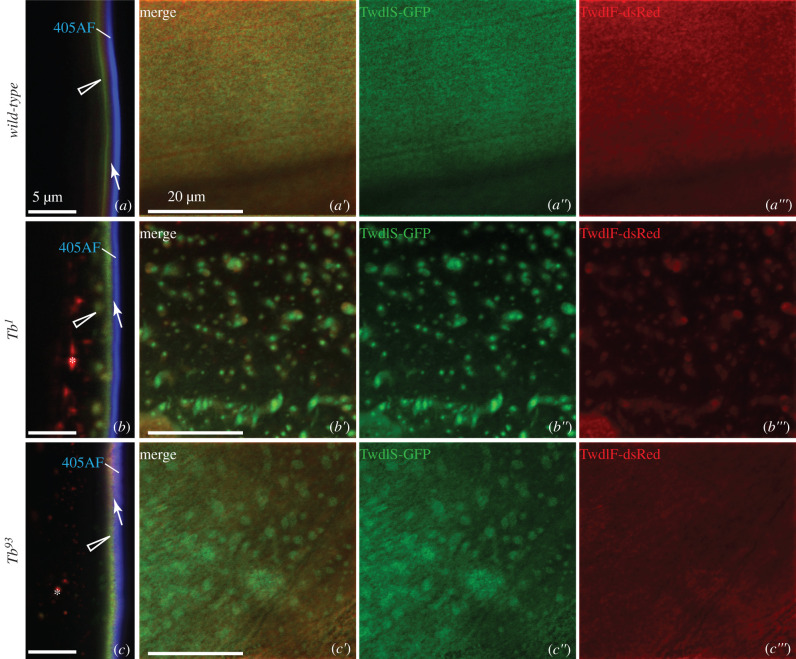
Localization of TwdlS-GFP and TwdlF-dsRed in the cuticle of wild-type, Tb^1^ and Tb^93^ third instar larvae. (*a*) TwdlF-dsRed (red, arrow) and TwdlS-GFP (green, triangle) form two separate layers in the cuticle of WT larvae in perfect optical cross-sections. TwdlF localizes just below the envelope (blue) and TwdlS-GFP under the TwdlF layer (*a*: lateral view, *a*′–*a*″′: top views with separated channels). In the *Tb^1^* larvae both proteins (TwdlF-dsRed red, arrow; TwdlS-GFP green, triangle) are partially mislocalized, forming aggregates in the lower cuticular layer as seen in optical cross-sections (*b*). Aggregates are well visible in successive top views (*b*′–*b*″′). In the *Tb^93^* larvae, there is no decent stratification of TwdlS-GFP and TwdlF-RFP in optical cross-sections (*c*). Only TwdlS-GFP (green, triangle) forms aggregates. This is best evident in top views of the same larva (*c*′–*c*″′). Asterisks label intracellular TwdlF-RFP particles.

**Figure 4.**
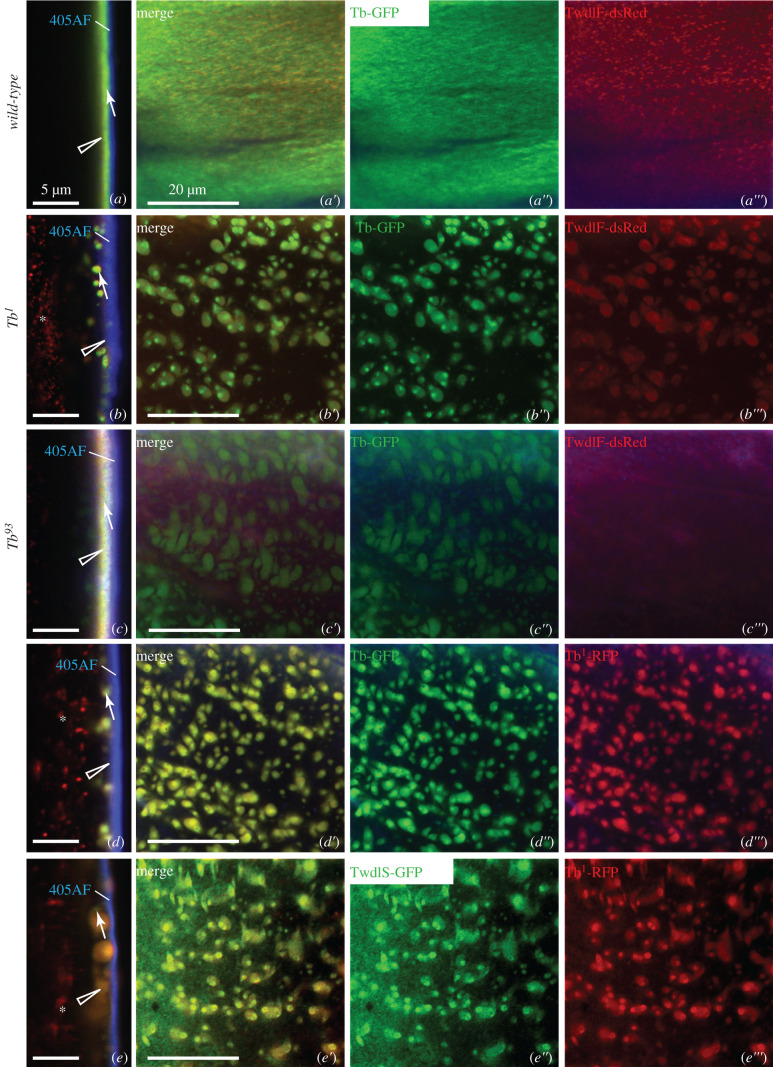
Localization of Tb-GFP, TwdlS-GFP and TwdlF-dsRed in the cuticle of wild-type, Tb^1^ and Tb^93^ third instar larvae. (*a*) In the cuticle of wild-type larvae, Tb-GFP (green, arrow) and TwdlF-dsRed (red, triangle) overlap (*a*: lateral view, *a*′–*a*″′: top views with separated channels). In the cuticle of *Tb^1^* larvae both proteins are partially aggregated (*b–b*″′). In the cuticle of *Tb^93^* larvae Tb-GFP forms aggregates (triangle), while TwdlF-dsRed does not (*c–c*″′). Aggregates are better visible in top views of the same larva (*c*′–*c*″′). In the cuticle of the larvae with two additional copies of RFP-tagged, mutated Tb protein (Tb1-RFP, red), the non-mutated Tb-GFP form (green) binds to the aggregates formed by Tb^1^-RFP (*d–d*″′). TwdlS-GFP protein (green) joins the Tb^1^-RFP aggregates as well (*e–e*″′).

In order to determine the sub-cuticular localization of Twdl proteins, we analysed the distribution of the three fluorescent-tagged Twdl proteins (i.e. TwdlS-GFP, TwdlF-dsRed and Tb-GFP) in the cuticle of live L3 larvae by confocal microscopy (figures 2–4). We used the auto-fluorescence of the cuticle surface excited with a 405 nm laser that represents the envelope [12] and the tagged procuticle markers Cuticle Protein R&R 67Fa1 (Cpr67Fa1-dsRed) and Vermiform (Verm-RFP) as landmarks. GFP-conjugated TwdlS was located between the envelope and the broad Cpr67Fa1-dsRed (figure 2*a–a*″′) or Verm-RFP (figure 2*d–d*″′) region marking the procuticle. When the optical cross-section was perfect, TwdlF-dsRed was detected in a layer above the TwdlS-GFP with partially overlapping signal, but below the envelope (figure 3*a*). Due to living larvae, a perfect optical cross-section was not always possible (figure 2*a*). Tb-GFP, by contrast, always overlapped with TwdlF-dsRed (figure 4*a*). We conclude that these Twdl proteins belong to a region between the envelope and the procuticle (i.e. the epicuticle possibly subdividing it into distinct horizontal domains).

**Figure 5.**
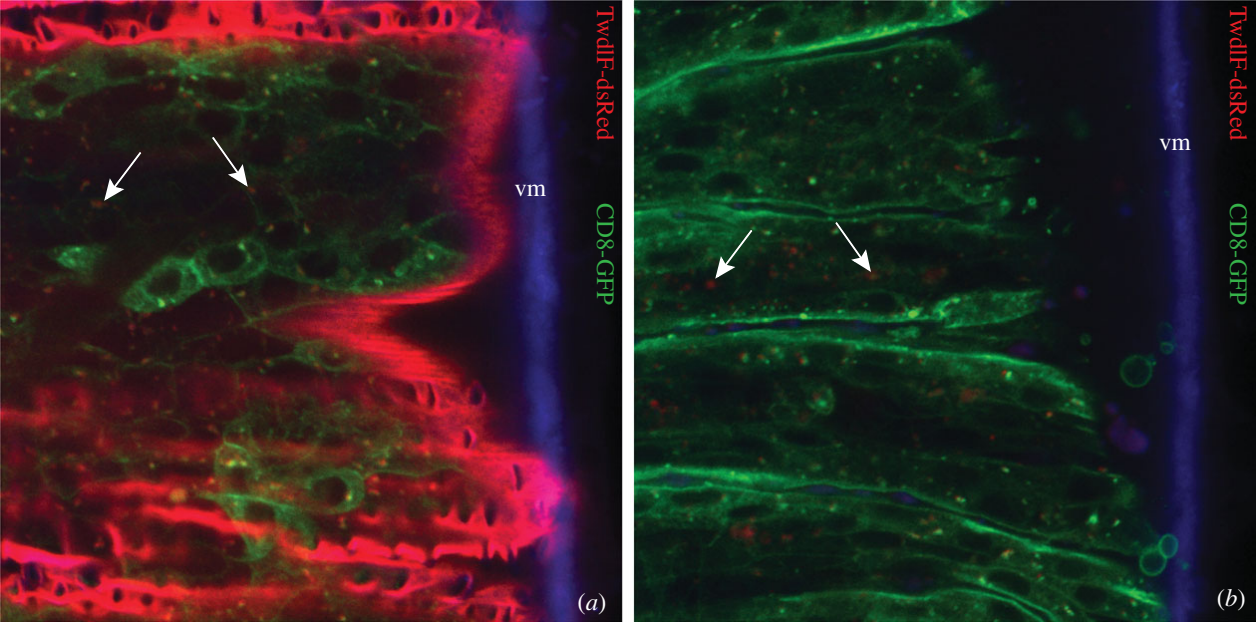
Localization of TwdlF-dsRed in WT and phm mutant larvae. (*a*) In the wild-type first instar larvae before hatching the signal of TwdlF-dsRed (red) is strong in the entire cuticle (green: ubiquitously produced membrane-binding CD8-GFP). Occasionally, dot-shaped red structures coincide with dot-shaped green structures inside the cell, probably representing secretory vesicles (arrows). (*b*) In *phantom* (*phm*) mutants, expression of TwdlF-dsRed is very low with a weak signal inside the cells probably corresponding to secretory vesicles (arrows). vm: vitelline membrane (blue) showing auto-fluorescence.

To test this conclusion, we monitored the expression of TwdlF-dsRed in embryos deficient for ecdysone that we had previously shown to be necessary for epicuticle differentiation [13]. In wild-type embryos, TwdlF-dsRed was detected in the entire larval cuticle (figure 5). In embryos mutant for *phantom* (*phm*) that codes for a P450 enzyme acting in the ecdysone biosynthesis pathway, TwdlF-dsRed was hardly expressed. Sporadically, dots of an RFP signal were found within epidermal cells. This finding is consistent with our conclusion that Twdl proteins localize to the epicuticle. Moreover, this result also indicates that activation of the *twdlF* promoter depends on ecdysone signalling.

### 2.3. Twdl proteins bind selectively to aggregates formed in Tb mutant larvae

To unravel the role of Twdl proteins in epicuticle formation and structure, we analysed the distribution of the tagged Twdl proteins in *Tb* mutant, i.e. *Tb^1^* and *Tb^93^* larvae. In *Tb^1^* larvae, TwdlS-GFP (figure 2*b–b*″′, *e–e*″′), TwdlF-dsRed (figure 3*b–b*″′) and Tb-GFP (figure 4*b–b*″′) were partially localized correctly in the epicuticle and partially aggregated in lower regions of the cuticle. In the cuticle of *Tb^93^* larvae, the signal of aggregated TwdlS-GFP (figures 2*c–c*″′, 3*f–f*″′) and Tb-GFP (figure 4*c–c*″′) was weaker compared with the signal in the *Tb^1^* mutant larvae, while TwdlF-dsRed (figure 3*c–c*″′) did not associate with the aggregates.

In order to find out whether the origin of the aggregates may be a mutated Tb protein, we compared the distributions of non-mutated, GFP-tagged and mutated, RFP-tagged versions of Tb, Tb-GFP and Tb^1^-RFP [14], respectively, in the cuticle of live L3 larvae. Tb^1^-RFP formed aggregates in the cuticle of these larvae (figure 4*d*,*e*). Co-expressed Tb-GFP was recruited to these aggregates (figure 4*d–d*″′). To test whether other Twdl proteins might be part of the Tb^1^-RFP aggregates, we co-expressed Tb^1^-RFP with TwdlS-GFP. The TwdlS-GFP signal overlapped with the signal of the Tb^1^-RFP aggregates (figure 4*e–e*″). We conclude that mutated Tb forms aggregates within the cuticle, which are able to recruit non-mutated Twdl proteins including Tb itself, TwdlD, TwdlF and TwdlS.

### 2.4. Tb^93^ is a twdlL allele

The differences in the localization of TwdlF-dsRed, TwdlS-GFP and Tb-GFP in both *Tb^1^* and *Tb^93^* alleles suggest that this discrepancy may be a consequence of different mutations in the *Tb* gene. Indeed, the phenotypes caused by these two alleles differ also between the larval stages. *Tb^1^* larvae start to show the squat phenotype at the L2 larval stage, while *Tb^93^* do so already at L1 (electronic supplementary material, figure S1).

The *Tb^1^* allele carries a deletion removing residues 167–190 of the protein including 18 amino acids within the DUF 243 domain [11]. Sequencing of the *Tb* gene in the *Tb^93^* genome revealed no changes in the Tb protein sequence (data not shown). We reckoned, therefore, that *Tb^93^* is not an allele of *Tb*. To test this notion, we sought to recombine the *Tb^1^* and *Tb^93^* alleles on one chromosome arguing that recombination would underline that these mutations affect different loci. For this purpose, in a population issued from a cross of *Tb^1^* and *Tb^93^* heterozygous flies segregating both mutations in trans, we screened for larvae that did not show the squat phenotype. We isolated four wild-type larvae in a population of 5860 larvae suggesting that the mutations affect different loci and that these loci are roughly 0.1 cM apart. Based on this result, we sequenced other *twdl* genes in the *twdl* cluster on the right arm of chromosome 3 in *Tb^93^* animals in order to identify the mutation responsible for the phenotype. Indeed, we found a missense mutation in the sequence coding for a conserved amino acid of the DUF 243 domain in another *Twdl* gene, *TwdlL* (electronic supplementary material, figure S2). In order to confirm that the *Tb^93^* phenotype is caused by the mutation in the *TwdlL* gene, we expressed a hairpin RNA directed against the *twdlL* gene in the background of *Tb^93^*. The hairpin RNA abolished the squat phenotypes of *Tb^93^* larvae and pupae. By contrast, hairpin RNA against *TwdlL* did not cause any changes in the phenotype of *Tb^1^* animals. As a control, we also tested several hairpin RNAs against other *twdl* genes in the *Tb^93^* background (table 1). No phenotype changes were observed in any of these crosses. We conclude that *Tb^93^* is a dominant allele of the *twdlL* gene (henceforth *TwdlL^93^*). Taken together, Tb and TwdlL are both needed for correct epicuticle formation.

**Table 1.** RNAi lines used in this study. Hairpin RNA constructs against *twdl* transcripts in the *twdl* cluster on chromosome 3 (*twdlJ, L, O* and *S*, see electronic supplementary material, figure S10) and outside this cluster (*twdlW* and *Z*) expressed in a *Tb^93^*/*TwdlL^93^* background to possibly suppress the tubby phenotype. These constructs were chosen because, according to the VDRC database, in contrast with all other hairpin constructs against *twdl* transcripts, they do not have off-targets.

gene	VDRC ID
*twdlL*	108513 KK
*twdlO*	107686 KK
*twdlJ*	103260 KK
*twdlW*	101966 KK
*twdlZ*	100874 KK
*twdlS*	101176 KK
*twdlS*	2677 GD

### 2.5. Twdl aggregates constitute an ectopic epicuticle immersed in the procuticle

**Figure 6.**
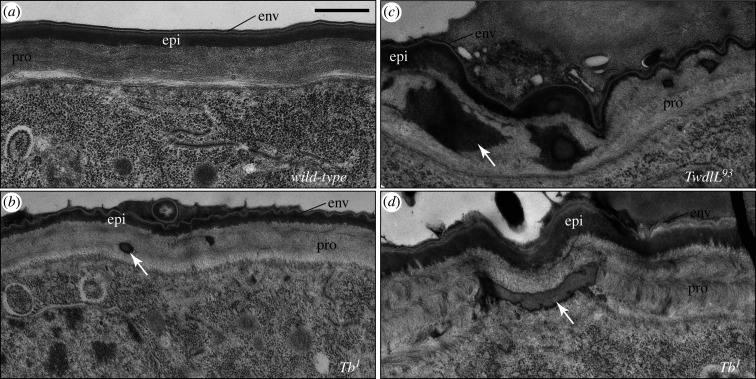
In Tb mutant larvae, Twdl proteins aggregate in the procuticle. (*a*) The wild-type first instar larval cuticle consists of three composite horizontal layers: the envelope (env), the epicuticle (epi) and the procuticle (pro). (*b*) The cuticle of *Tb^1^* mutant first instar larvae is stratified as the wild-type cuticle. Occasionally, electron-dense material is found in the procuticle (arrow). (*c*,*d*) At later stages, large electron-dense material accumulates within the procuticle in *Tb^1^* and *Tb^93^* mutant larvae. Electron micrographs. Scale bar, 500 nm.

The aggregates visible on optical cross-sections of the cuticle shown in figures 2–4 do not localize to the expected region of the epicuticle just below the surface but scattered along the *z*-axis of the cuticle. To precisely localize these aggregates, we analysed the ultrastructure of the *Tb^1^* and *TwdlL^93^* larval cuticle by transmission electron microscopy (figure 6). We observed electron-dense aggregates immersed within the procuticle. These aggregates were absent in the cuticle of wild-type larvae.

The interpretation that Twdl aggregates localize to the procuticle is supported by localization experiments with TwdlS-GFP and the tagged chitin-binding, procuticular proteins Cpr67Fa1-RFP and Verm-RFP co-expressed in *Tb^1^* (figure 2*b–b*″′,*e–e*″′) and *TwdlL^93^* (figure 2*c–c*″′,*f–f*″′) mutant larvae. TwdlS-GFP aggregates were embedded in a region marked by Cpr67Fa1-RFP and Verm-RFP. Taken together, we conclude that Twdl proteins form an ectopic epicuticle within the procuticle of *Tb^1^* and *TwdlL^93^* larvae recruiting non-mutated Twdl proteins (i.e. TwdlS-GFP) from the epicuticle.

### 2.6. Distribution of Twdls in hairs differs from their distribution in the naked cuticle

**Figure 7.**
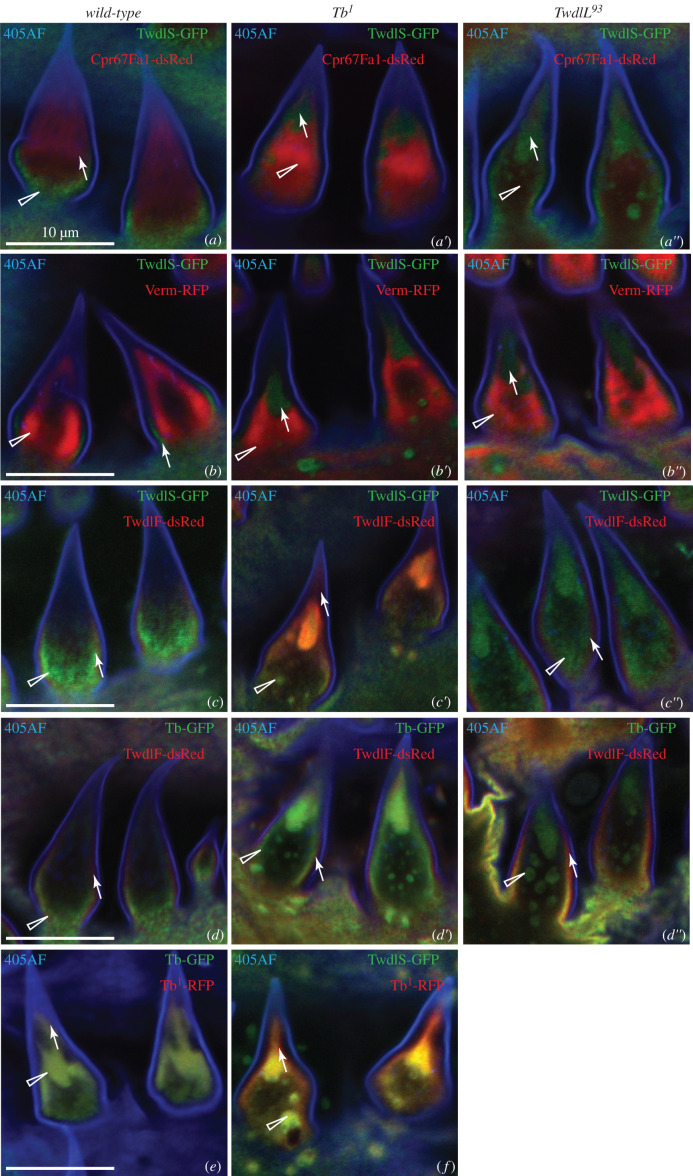
Localization of Twdl fluorescent proteins in the hairs of wild-type and Tb mutant larvae. In the dorsal hairs of wild-type larvae TwdlS-GFP (green) forms a layer under the auto-fluorescent line (blue) at the bases of the hairs but does not reach their tip (*a*,*b*). Cpr67Fa1-dsRed (*a*) and Verm-RFP (*b*) are localized in the centre of hairs (both red). In the hairs of *Tb^1^* and *Tb^93^* larvae, TwdlS-GFP accumulates at the tips and forms smaller aggregates in the middle of the hair, while Cpr67Fa1-dsRed and Verm-RFP are localized in the centre, excluding the TwdlS areas (*a*′–*a*″; *b*′–*b*″). TwdlF-dsRed (red) forms a layer between the auto-fluorescent layer and TwdlS-GFP layer (405AF) in the hairs of wild-type larvae (*c*). In the hairs of *Tb^1^* larvae stratification under the envelope seems to be retained, while at the hair tip and in the aggregate proteins overlap (*c*′). In *Tb^93^* larvae stratification is retained and only TwdlS-GFP is mislocalized (*c*″). Localization of Tb-GFP (green) in the hairs of wild-type, *Tb^1^* and *Tb^93^* larvae is similar to TwdlS-GFP (*d–d*″); these larvae also expressed TwdlF-dsRed. Mutated Tb^1^-RFP (red) form attracts unmutated Tb-GFP (*e*, green) and TwdlS-GFP (*f*, green).

Separation of the pro- and epicuticle in bristles and hairs in the *D. melanogaster* larvae is less pronounced than in the naked cuticle [15]. For this reason, we had a closer look at the distribution of Twdl proteins in the dorsal hairs in third instar larvae. The organization of the epicuticle in dorsal hairs of wild-type larvae was similar to the naked cuticle: Twdl proteins form adjacent layers under the blue auto-fluorescent layer (figure 7*a*–*d*; electronic supplementary material, figure S3). The procuticle was located in the middle of a hair and reached its tip. In the *Tb^1^* and *TwdlL^93^* mutant background, TwdlS-GFP (figure 7*a*′*c*″), TwdlF-dsRed (figure 7*c*′*d*″) and Tb-GFP (figure 7*d′*,*d*″) accumulated at the tips of the hair. The procuticle marked with Cpr67-dsRed (figure 7*a–a*′) and Verm-RFP (figure 7*b–b*″) still remained in the middle of the hair, except for the tip, so that these two layers (Twdl and procuticle) did not overlap. Thus, mutations in *Tb^1^* and *TwdlL^93^* cause a lateral shift of Twdl protein localization from more basal sites of the hair to its tip.

### 2.7. Mislocalization of the dityrosine layer in Tb mutant larvae

**Figure 8.**
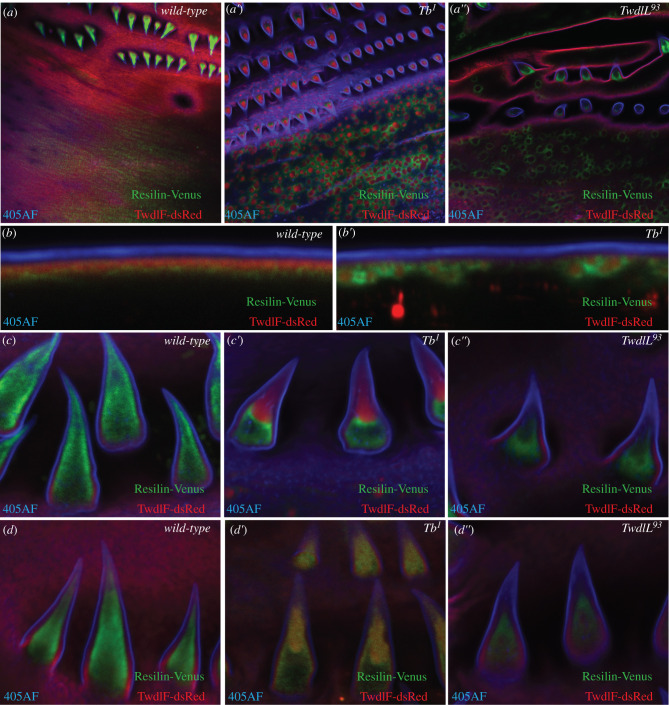
Mislocalization of the dityrosine layer in Tb mutant larvae. In the *wild-type* third instar one-day-old larvae Resilin-Venus (green) is plainly distributed in the whole cuticle (*a*, top view on the larval segment), while in the *Tb^1^* (*a*′) and *Tb^93^* (*a*″) mutant larvae it is attracted to the aggregates and encircles them (red: TwdlF-dsRed, blue: 405 nm-induced auto-fluorescent envelope signal 405AF). In the optical cuticle cross-section of the *wild-type* one-day old larvae Resilin-Venus localizes just below the TwdlF-dsRed layer (*b*), while in *Tb^1^* mutant larvae it predominantly surrounds the aggregates visualized by TwdlF-dsRed (*b*′). In the dorsal hairs of the young *wild-type* third instar larvae just after moutling Resilin-Venus forms a uniform layer below the TwdlF-dsRed layer in the whole hair, while the hair core contains less signal (*c*). In the hairs of the respective *Tb^1^* (*c*′) and *Tb^93^* (*c*″) mutants, Resilin-Venus appears to be normal. TwdlF-dsRed localizes in the presumptive hair procuticle. In one-day-old third instar *wild-type* larvae localization of Resilin-Venus is unchanged in comparison with the third instar larvae just after hatching (*d*), while in one-day-old *Tb^1^* (*d*′) and *Tb^93^* (*d*″) larvae Resilin-Venus is not localized at the hair tip anymore. TwdlF-dsRed localization is unchanged compared with younger larvae.

Recently, we showed that the contact zone between the epicuticle and the procuticle is marked by dityrosinylated proteins in *D. melanogaster* larvae [10]. In the *wild-type* third instar one-day-old larvae, Resilin-Venus representing the dityrosinylated layer localized beneath the TwdlF-dsRed layer (figure 8*a*,*b* and electronic supplementary material, figure S4). In the *Tb^1^* and *TwdlL^93^* mutant larvae at the same stage, Resilin-Venus was attracted to the aggregates and encased them (figure 8*a′*,*a*″,*b*′). In the dorsal hairs of the young *wild-type* third instar larvae just after moulting Resilin-Venus occupied the space below the TwdlF-dsRed layer in the dorsal hair (figure 8*d*), while in the hairs of the *Tb^1^* and *TwdlL^93^* mutant larvae, it accumulated also at the hair tips, where the aggregated Tweedle proteins occurred (figure 8*d*′,*d*″). In one-day-old third instar wild-type larvae localization of Resilin-Venus was unchanged compared with young third instar larvae (figure 8*c*), while in one-day-old *Tb^1^* and *TwdlL^93^* mutant larvae Resilin-Venus was not localized at the hair tips anymore but encased the Twdl aggregates at the hair tip (figure 8*c*′,*c*″). Thus, Twdl proteins dictate the localization and the shape of the dityrosinylated layer at the epicticle–procuticle interface.

### 2.8. Non-cell autonomous localization of the aggregated Twdls in the cuticle

In our live localization experiments, we noted that, as reported, TwdlD-RFP was expressed in a striped pattern in wild-type larvae. Dorsal hairs were lacking TwdlD-RFP. In the cuticle of *Tb^1^* and *TwdlL^93^* larvae (electronic supplementary material, figure S5), the Twdl aggregates were visible in the areas of dorsal hairs including the tips of hairs. Two alternative scenarios may explain this observation. Either the expression pattern of *twdlD* was changed in *Tb^1^* and *TwdlL^93^* larvae, or, in addition to the vertical, the lateral mobility of the TwdlD within the cuticle was enhanced in these larvae. In order to distinguish between these two possibilities, we monitored the expression of a nuclear-binding RFP under the control of the *TwdlD* promoter (TwdlD > RFP-NLS) in wild-type, *Tb^1^* and *TwdlL^93^* larvae. In all three cases RFP-NLS was detected in a striped pattern in the epidermis of developing embryos and larvae. This finding indicates that there were no changes in the expression pattern of *twdlD*. Thus, the occurrence of the aggregates in the whole cuticle was due to the lateral spreading of the protein in the extracellular space.

### 2.9. Epidermal cell shape is altered in Twdl mutant larvae

Compared with wild-type larvae, *Tb^1^* and *TwdlL^93^* mutant larvae are wider, but shorter (electronic supplementary material, figure S1). To study to what extent the body shape difference is reflected at the cellular level in *Tb^1^* and *TwdlL^93^* larvae, we examined the shapes of their epidermal cells visualized by the membrane bound CD8-GFP protein. In all larvae tested, the number of cells and their average area were unchanged, but the shape of the cells was significantly altered in *Tb^1^* and *TwdlL^93^* larvae (electronic supplementary material, figures S6 and S7). These cells were shorter along the anterior–posterior axis and longer along the dorsoventral axis of the animal as compared with cells of wild-type larvae.

### 2.10. Movement capabilities of the Tb^1^ and TwdlL^93^ larvae are normal

An intact exoskeleton is a prerequisite for insect locomotion. To find out whether the altered body shape of *Tb^1^* and *TwdlL^93^* larvae affects crawling efficiency, we measured the ratio of the body length in the most stretched to the most contracted state of young third instar wild-type and *Tb^1^* and *TwdlL^93^* larvae (electronic supplementary material, figure S6). We observed there was no significant difference between the ratios of the wild-type and *Tb^1^* and *TwdlL^93^* larvae. We also investigated whether the barrel-like larval shape limits the crawling capabilities of the *Tb^1^* and *TwdlL^93^* larvae. For this purpose, we measured the height on which larvae formed pupae on the vial wall. We found that in all cases, the wild-type, *Tb^1^* and *TwdlL^93^* homozygous larvae, the average pupariation height was comparable (electronic supplementary material, figure S8). Taken together, we conclude that the stretchiness of the cuticle of the *Tb^1^* and *TwdlL^93^* larvae and their movement efficiency are unchanged.

### 2.11. Basal cuticular ridges are disorganized in Tb^1^ and TwdlL^93^ mutant larvae

**Figure 9.**
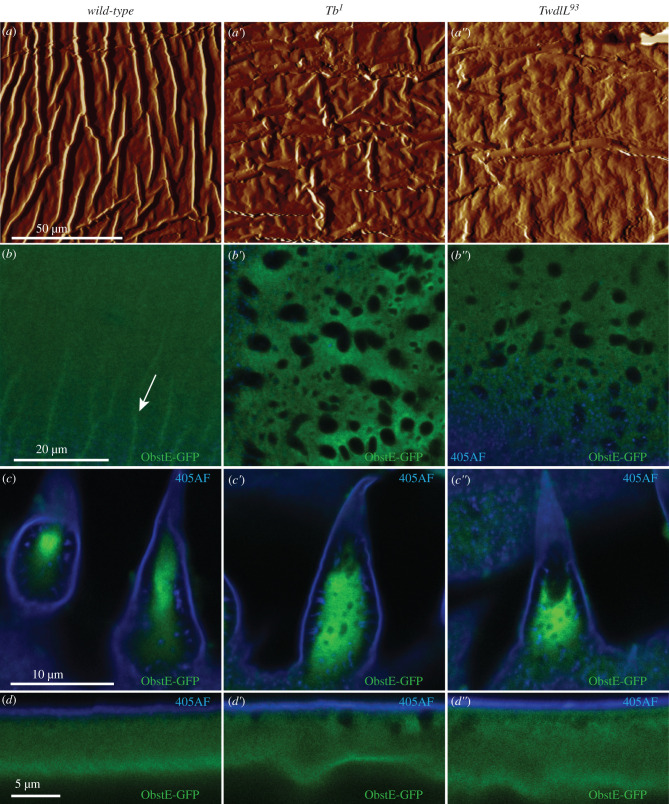
The structure of procuticular ridges but not the localization of Obstructor-GFP is changed in Tb mutant larvae. The internal cuticular surface of the wild-type L3 larvae scanned by the atomic force microscope shows longitudinal ridges running parallel to the anterior–posterior axis (*a*). In *Tb^1^* (*a*′) and *TwdlL^93^* mutants (*a*″), the structure of the ridges is disrupted, they run in different directions and seem to be flatter. GFP-tagged chitin-binding protein Obstructor-E (Obst-E-GFP, green) is plainly distributed in the whole procuticle of the naked cuticle (*b*: the top view of the dorsal cuticle; *d*: the lateral view; blue: auto-fluorescence of the external cuticular envelope, 405AF) and in the centre of the dorsal hairs of wild-type larvae (*c*). On the top view the cuticular ridges are discernible (*b*, marked with arrow). In the *Tb^1^* (*b*′–*d*′) and the *Tb^93^* larvae (*b*″–*d*″) Obst-E-GFP is still plainly distributed in the procuticle, excluding the epicuticular aggregates.

The cuticular protein Obstructor-E (Obst-E) is needed for ridge formation at the interface between the procuticle and the apical plasma membrane of epidermal cells [16]. These ridges are missing in *obst-E* mutant larvae that by consequence do not contract during pupariation. The question is whether these ridges are altered in *Tb^1^* and *TwdlL^93^* mutant larvae. We analysed the basal site of the procuticle in wild-type, *Tb^1^* and *TwdlL^93^* late third instar larvae by atomic force microscopy (AFM). The procuticle of wild-type larvae formed long convexities along the antero-posterior axis of the larva (figure 9*a*). The inner cuticular surface of *Tb^1^* and *TwdlL^93^* larvae formed convexities that were comparably flat with random orientation (figure 9*a*′,*a*″). Hence, Twdl proteins are needed for correct orientation of the basal procuticular ridges.

In confocal imaging experiments, we observed that the cuticular aggregates in *Tb^1^* and *TwdlL^93^* larvae were in contact with the apical site of the epidermal cells (electronic supplementary material, figure S9). Thus, the effect of Twdl proteins on apical ridge formation is either direct or indirect.

### 2.12. Localization of the Obst-E does not depend on Twdl function

Both, Twdl and Obst-E proteins influence the formation of basal cuticular ridges. To test whether Twdl proteins may influence Obst-E localization and function, we monitored Obst-E-GFP localization in wild-type, in *Tb^1^* and *TwdlL^93^* late third instar larvae. In non-squat L3 larvae Obst-E-GFP was uniformly localized in the procuticle (figure 9*b*–*d*). In *Tb^1^* and *Twdl^93^* larvae Obst-E-GFP localization was unchanged and did not bind to the Twdl aggregates (figure 9*b*′-*d*″). We conclude that the localization of Obst-E does not depend on the function of Twdl proteins.

## 3. Discussion

ECMs and the ECM-producing cells adopt a concerted shape that is potentially important for tissue function. The insect integument consisting of the epidermis and the apical cuticle, for instance, conceivably plays a key role in body shape determination. The two Twdl-class cuticle proteins Tb and TwdlD have been shown to be involved in this process in *D. melanogaster* [11].

### 3.1. Twdl proteins form two-dimensional sheets within the epicuticle

Live imaging experiments with fluorescent-tagged proteins using CLSM reveal that Tb, TwdlF and TwdlS proteins form two two-dimesional adjacent horizontal sheets (i.e. the TwdlF and the Tb/TwdlS sheets underneath the surface and above the procuticle that is characterized by the presence of dityrosinylated proteins). This localization indicates that Twdl proteins possibly belong to the epicuticle. In agreement with this interpretation, in ecdysone biosynthesis mutants where the epicuticle is absent [13], *twdl* gene expression is strongly reduced. We also conclude that the uniform appearance of the epicuticle in electron micrographs probably does not reflect the stratified organization revealed by fluorescence CLSM. Thus, the epicuticle may be a more complex structure than supposed by mere ultrastructure analysis.

Dominant mutations in *twdl* genes provoke mislocalization of the mutated proteins and their accumulation as three-dimensional aggregates within the procuticle. Non-mutated Twdl proteins are incorporated into these structures. Based on these data we assume that Twdl proteins interact with each other. These observations argue that the mutated Twdl protein sequence loses its ability to form a flat two-dimensional ECM but recruits normal and mutated protein sequences to form ectopic three-dimensional aggregates without losing the ability to self-assemble. The recruitment, however, is selective (i.e. not all Twdl proteins tested are attracted to these aggregates). In addition, mutant Twdl proteins attract dityrosinylated proteins from the epicuticle–procuticle interface that had previously been shown to have an important role in cuticle organization and function [10,17]. This supports the notion that Twdl protein polymers are responsible for the formation and orientation of the adjacent dityrosine sub-layer including Resilin that is assumed to confer elasticity.

In summary, the epicuticle consists of polymers of Twdl proteins that partition this layer into two two-dimensional horizontal sheets by self-assembly.

### 3.2. Squat body shape as a consequence of body wall tension changes

Our data underline that Twdl proteins play a key role in body shape determination or maintenance. This function might rely on their localization and function within the cuticle. Alternatively, the dominant phenotype caused by *Twdl* mutations, however, suggests that the defects may be neomorphic (i.e. they might be unrelated to the normal function of these proteins). Together, three possible scenarios can explain the mechanism of Twdl protein function in body shape implementation: (1) the ‘cytoplasmic’, (2) ‘epidermal–cuticular interface’ and (3) ‘cuticle’ scenarios.

The ‘cytoplasmic’ theory relies on mutated Twdl proteins that fail to be transported to the cuticle but accumulate in cytoplasmic structures, probably vesicles, during cuticle formation when massive secretion and vesicle sorting occur [5,18]. Accumulation of Twdl-vesicles may perturb plasma membrane dynamics, and thereby cause loss of correct cell shape along the antero-posterior axis. The altered cell shape would, in turn, influence the body shape.

The alternative ‘epidermal–cuticular’ theory relies on the extracellular Twdl aggregates that are in close contact with the apical surface of the epidermal cells. According to this theory, these aggregates in the procuticle are responsible for the dis-organization of the regular ridges that run along the antero-posterior axis in the epidermal–cuticular interface [16]. These ridges have recently been shown to depend on the presence of the procuticular protein Obst-E that controls longitudinal contraction and lateral expansion of the L3 larvae during pupariation. The deletion of *obst-E* causes flattening of the ridges and the formation of longer and thinner pupae. In *twdl* mutant larvae, Obst-E localization appears to be normal. We, therefore, reckon that body shape changes in *twdl* mutant animals are independent of Obst-E function. As a consequence of ridge mis-orientation in *twdl* mutant larvae, however, the epidermal cells lose their longitudinal antero-posterior direction and adopt a shape with random orientation. Accordingly, the change of orientation preference of epidermal cells may be responsible for the overall shorter but thicker body shape.

**Figure 10.**
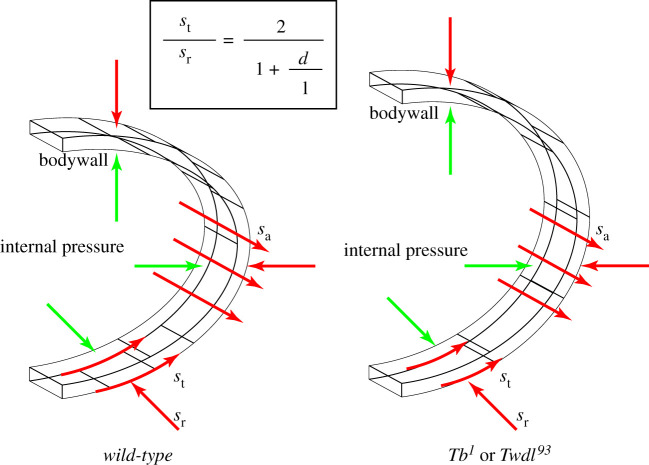
Barlow's law and the role of Twdls: a model. According to Barlow's law, the wall of a pipe experiences three types of internal stresses: the radial stress *s*_r_, the tangential stress *s*_t_ and the axial stress *s*_a_. Depending the physical properties of the wall and the internal pressure, an equilibrium is attained (left scheme). Weakening of the wall at a constant internal pressure causes a radial expansion at the expense of elongation (right scheme). This is what happens in *twdl* mutant larvae, if we consider the larval body as a pipe.

The third, ‘cuticle’ theory considers that the depletion of either the epicuticle itself or the epicuticle–procuticle interface might be the reason for the aberrant body shape in *twdl* mutant animals. In this view, the Twdl polymers and/or the dityrosynilated proteins of the epicuticle–procuticle interface confer the elastic forces resisting the internal hydrostatic pressure. A thin epicuticle and/or epicuticle–procuticle sub-layer may be insufficient to withstand these forces. According to the formula of Barlow, a weakened wall of a closed pipe or cylinder would allow radial rather than longitudinal expansion of the object (figure 10). In analogy, due to a weakened cuticle and assuming a normal hydrostatic pressure, *twdl* mutant larvae become thick and short. Epidermal cells would, in this scenario, passively follow cuticle stretching in the lateral direction.

In any case, body shape change in *twdl* mutant larvae does not affect locomotion efficiency. Importantly, the *twdl* mutant phenotype enables us to distinguish body shape and locomotion as distinct functions of the cuticle that are not necessarily linked.

### 3.3. Twdl evolution

The *D. melanogaster* genome encodes 27 *twdl* genes. Different combinations of the respective proteins in different body regions allow establishment of different types of epicuticles, thereby, conforming with our ‘cuticle’ theory, probably influencing the physical properties of the cuticle. So far so good. By contrast to *D. melanogaster* some insects such as the bedbug *Cimex lectularius* or the honeybee *Apis mellifera* do only have two or three copies of *twdl* in their genome [19]. How is epicuticle complexity that we encounter in *D. melanogaster* achieved in these species? We can only speculate that, testified by the varying number of *twdl* genes, the epicuticle is a fast-evolving structure and therefore reckon that other types of epicuticular proteins may contribute to its construction to accommodate its different functions in species with only a few *twdl* genes.

## 4. Material and methods

### 4.1. Fly husbandry

Flies were kept in cages with apple juice agar plates with yeast, from which embryos and larvae were collected. Embryonic stages were recognized according to the gut morphology described by Hartenstein & Campos-Ortega [20]. Homozygous mutants non-carrying any GFP- or YFP-constructs were identified from the rest of the embryos that were heterozygous or homozygous for the balancer chromosome expressing YFP or GFP (Dfd:YFP or Kr:GFP). Collected embryos were dechorionated in chlorine bleach diluted 1:1 in tap water, manually freed from the vitelline membrane or left in the vitelline membrane, subsequently mounted in Voltalef 10S oil (VWR Chemicals) and observed by microscopy.

Transgenic flies harbouring constructs were generated by the BestGene Inc (USA) company. The constructs used are: *knkp*:*cpr67Fa*-*rfp* with the promoter of the *knk* gene [13] upstream of the coding region of *cpr67Fa* fused to the open reading frame of *rfp* in pW8; TwdlD-RFP-NLS with the coding regions of *twdlD* and *rfp-nls* fused together and downstream of the *twdlD* promoter [11] in pW8, Tb-GFP and TwdlS-GFP from the Flyfos library (electronic supplementary material, figure S10) [21], Tb1-RFP [14], TwdlF-dsRed and TwdlD-dsRed [11], UAS:Verm-RFP [22], UAS-Resilin-Venus [10] and Obst-E-GFP [16]. The Gal4 line to drive UAS:*Verm*-*Rfp* and UAS-Resilin-Venus expression was *daughterless*:Gal4.

### 4.2. Molecular biology

In order to identify a mutation in *TwdlL* gene standard PCR reaction and sequencing were performed. The primers used for the amplification and sequencing of the *twdl* gene are listed in electronic supplementary material, table S1.

### 4.3. Genetics

In order to downregulate transcript levels of *twdl* genes in a *Tb^93^*/*TwdlL^93^* background, the RNAi technique combined with the UAS:Gal4 system was applied. Flies carrying a UAS:RNAi construct coding for a hairpin RNA directed against a corresponding *twdl* transcript were crossed to flies harbouring ubiquitously or epidermally expressed Gal4 (*daughterless:*Gal4 or *e22c*:Gal4, respectively). The larval and pupal phenotypes of the progeny were observed. The RNAi lines used in this work are listed in table 1 and were purchased from the VDRC (Vienna, Austria).

For confirmation that *Tb^1^* and *Tb^93^* mutations are not alleles of the same gene we performed a complementation test by crossing *Tb^1^* and *Tb^93^* flies with each other, subsequently crossing daughters (F1 females) with wild-type males and counting the number of the wild-type and *tubby* looking pupae.

### 4.4. Microscopy and image preparation

For live imaging, larvae were anaesthesized with ether, mounted in halocarbon 700 or 50% glycerol and observed either by confocal laser scanning microscopy (CLSM, using Zeiss LSM 710, 780 and 880) or fluorescent binocular Leica M205 FA.

For imaging of the cuticle preparations, the larvae were mounted in Hoyer's medium (30 g gum arabic, 50 ml distilled water, 200 g chloral hydrate, 20 g glycerol, mixed 1 : 1 with lactic acid), kept overnight at 65°C and observed on a binocular Leica M205 FA. Transmission electron microscopy was performed following our extensive protocol published in 2010 [23]. For examination of the cuticular ridges, third instar larvae were digested in Hoyer's medium and their cuticles washed several times in distilled water. Afterwards they were stacked to the metal plate and their inner side was scanned by the atomic force microscope (Innova AFM, Bruker).

For figures’ preparation Adobe Photoshop CS3/CS6 and Adobe Illustrator CS4/CS6 software were used without changing initial microscope settings. For cell measurements, AxioVision Rel. 4.7 was used.

### 4.5. Body length measurements

Third instar larvae were placed on an agar plate and the movie of 10 steps (1 step = contraction and subsequent stretching) was made. Afterwards in every step the body in the most contracted and stretched state was measured and the difference between the shortest and the longest measurement of all 10 steps of 5 wild-type, homozygous *Tb^1^* and *Tb^93^* larvae was counted and compared.

### 4.6. Movement measurements

In total, 100 third instar wild-type, homozygous *Tb^1^*and *Tb^93^* larvae were placed into vials with agar food, 20 larvae of each kind in one vial. After pupariation of all larvae, the distance between the pupae on the vial wall and the food level was measured and the average of measurements of 20 larvae was determined. To compare the crawling efficiency between wild-type and *twdl* mutant larvae, five larvae of each genotype were filmed. The maximally stretched and contracted states of the body were determined and measured on images extracted from the movies.

## Supplementary Material

Suppl data

## Data accessibility

This article does not have additional data.

## References

[1] Kiani C, Chen L, Wu YJ, Yee AJ, Yang BB 2002 Structure and function of aggrecan. Cell Res. 12, 19–32. (10.1038/sj.cr.7290106) 11942407

[2] Horkay F 2012 Interactions of cartilage extracellular matrix macromolecules. J. Polym. Sci. B Polym. Phys. 50, 1699–1705. (10.1002/polb.23191) 23997426 PMC3755958

[3] Horkay F, Basser PJ, Hecht AM, Geissler E 2011 Hierarchical organization of cartilage proteoglycans. Macromo.l Symp. 306–307, 11–17. (10.1002/masy.201000115) PMC3615634 23565043

[4] Horkay F, Basser PJ, Hecht AM, Geissler E 2017 Structure and properties of cartilage proteoglycans. Macromol. Symp. 372, 43–50. (10.1002/masy.201700014) 29731595 PMC5931741

[5] Moussian B 2010 Recent advances in understanding mechanisms of insect cuticle differentiation. Insect. Biochem. Mol. Biol. 40, 363–375. (10.1016/j.ibmb.2010.03.003) 20347980

[6] Moussian B 2013 The arthropod cuticle. In Arthropod biology and evolution (eds Minelli A, Boxshall G, Fusco G), pp. 171–196. Berlin, Germany: Springer.

[7] Noh MY, Kramer KJ, Muthukrishnan S, Kanost MR, Beeman RW, Arakane Y 2014 Two major cuticular proteins are required for assembly of horizontal laminae and vertical pore canals in rigid cuticle of *Tribolium castaneum*. Insect. Biochem. Mol. Biol. 53, 22–29. (10.1016/j.ibmb.2014.07.005) 25042128

[8] Mun S, Noh MY, Dittmer NT, Muthukrishnan S, Kramer KJ, Kanost MR, Arakane Y 2015 Cuticular protein with a low complexity sequence becomes cross-linked during insect cuticle sclerotization and is required for the adult molt. Sci. Rep. 5, 10484 (10.1038/srep10484) 25994234 PMC4440208

[9] Shaik KS, Meyer F, Vazquez AV, Flotenmeyer M, Cerdan ME, Moussian B 2012 Delta-aminolevulinate synthase is required for apical transcellular barrier formation in the skin of the *Drosophila* larva. Eur. J. Cell Biol. 91, 204–215. (10.1016/j.ejcb.2011.11.005) 22293958

[10] Zuber R et al. 2019 The putative C-type lectin Schlaff ensures epidermal barrier compactness in *Drosophila*. Sci. Rep. 9, 5374 (10.1038/s41598-019-41734-9) 30926832 PMC6440989

[11] Guan X, Middlebrooks BW, Alexander S, Wasserman SA 2006 Mutation of TweedleD, a member of an unconventional cuticle protein family, alters body shape in *Drosophila*. Proc. Natl Acad. Sci. USA 103, 16 794–16 799. (10.1073/pnas.0607616103) PMC1636534 17075064

[12] Zuber R et al. 2018 The ABC transporter Snu and the extracellular protein Snsl cooperate in the formation of the lipid-based inward and outward barrier in the skin of *Drosophila*. Eur. J. Cell Biol. 97, 90–101. (10.1016/j.ejcb.2017.12.003) 29306642

[13] Gangishetti U, Veerkamp J, Bezdan D, Schwarz H, Lohmann I, Moussian B 2012 The transcription factor Grainy head and the steroid hormone ecdysone cooperate during differentiation of the skin of *Drosophila melanogaster*. Insect. Mol. Biol. 21, 283–295. (10.1111/j.1365-2583.2012.01134.x) 22458773

[14] Pina C, Pignoni F 2012 Tubby-RFP balancers for developmental analysis: FM7c 2xTb-RFP, CyO 2xTb-RFP, and TM3 2xTb-RFP. Genesis 50, 119–123. (10.1002/dvg.20801) 21913310 PMC3931234

[15] Moussian B, Seifarth C, Muller U, Berger J, Schwarz H 2006 Cuticle differentiation during *Drosophila* embryogenesis. Arthropod. Struct. Dev. 35, 137–152. (10.1016/j.asd.2006.05.003) 18089066

[16] Tajiri R, Ogawa N, Fujiwara H, Kojima T 2017 Mechanical control of whole body shape by a single cuticular protein Obstructor-E in *Drosophila melanogaster*. PLoS Genet. 13, e1006548 (10.1371/journal.pgen.1006548) 28076349 PMC5226733

[17] Fristrom D, Doctor J, Fristrom JW 1986 Procuticle proteins and chitin-like material in the inner epicuticle of the *Drosophila* pupal cuticle. Tissue Cell 18, 531–543. (10.1016/0040-8166(86)90019-4) 3092400

[18] Moussian B, Veerkamp J, Muller U, Schwarz H 2007 Assembly of the *Drosophila* larval exoskeleton requires controlled secretion and shaping of the apical plasma membrane. Matrix Biol. 26, 337–347. (10.1016/j.matbio.2007.02.001) 17360167

[19] Soares MP, Silva-Torres FA, Elias-Neto M, Nunes FM, Simoes ZL, Bitondi MM 2011 Ecdysteroid-dependent expression of the tweedle and peroxidase genes during adult cuticle formation in the honey bee, *Apis mellifera*. PLoS ONE 6, e20513 (10.1371/journal.pone.0020513) 21655217 PMC3105072

[20] Hartenstein V, Campos-Ortega JA 1985 The embryonic development of *Drosophila melanogaster*. Berlin, Germany: Springer-Verlag.

[21] Ejsmont RK, Sarov M, Winkler S, Lipinski KA, Tomancak P 2009 A toolkit for high-throughput, cross-species gene engineering in *Drosophila*. Nat. Methods 6, 435–437. (10.1038/nmeth.1334) 19465918

[22] Forster D, Armbruster K, Luschnig S 2010 Sec24-dependent secretion drives cell-autonomous expansion of tracheal tubes in *Drosophila*. Curr. Biol. 20, 62–68. (10.1016/j.cub.2009.11.062) 20045324

[23] Moussian B, Schwarz H 2010 Preservation of plasma membrane ultrastructure in *Drosophila* embryos and larvae prepared by high-pressure freezing and freeze-substitution. Drosophila Inf. Ser. 93, 215–219.

