# Supplementary material for: Tweedle proteins form extracellular two-dimensional structures defining body and cell shape in Drosophila melanogaster
Source: Open Biol. 2020 Dec 9;10(12):200214. doi: 10.1098/rsob.200214 (PMC7776580; doi:10.1098/rsob.200214)
Supplement: Suppl data [file rsob200214supp1.pdf]

Suppl. Fig. 1. Comparison of the body shape of the wild-type, Tb^1 and Tb^{93} larvae at three stages.

Hoyer's preparations of larval cuticles reveal that the first, second and third instar Tb^{93} larvae are decently shorter and thicker than the wild-type larvae (A). Second and third instar Tb^1 larvae are clearly shorter and thicker, but the first instar larvae are only a little bit shorter than the wild-type larvae (A). The shape differences between Tb and wild-type larvae become more visible at every subsequent stage. (B) The average ratio of length to the width of measured cuticle preps of the wild-type, $Tb^1$and Tb^{93} first, second and third instar larvae. The width and the length of 10 individuals were measured for each genotype. Differences between wild-type and mutant larvae are significant after a Student's T-test. The p-values for each pairwise comparison are indicated in the graph.

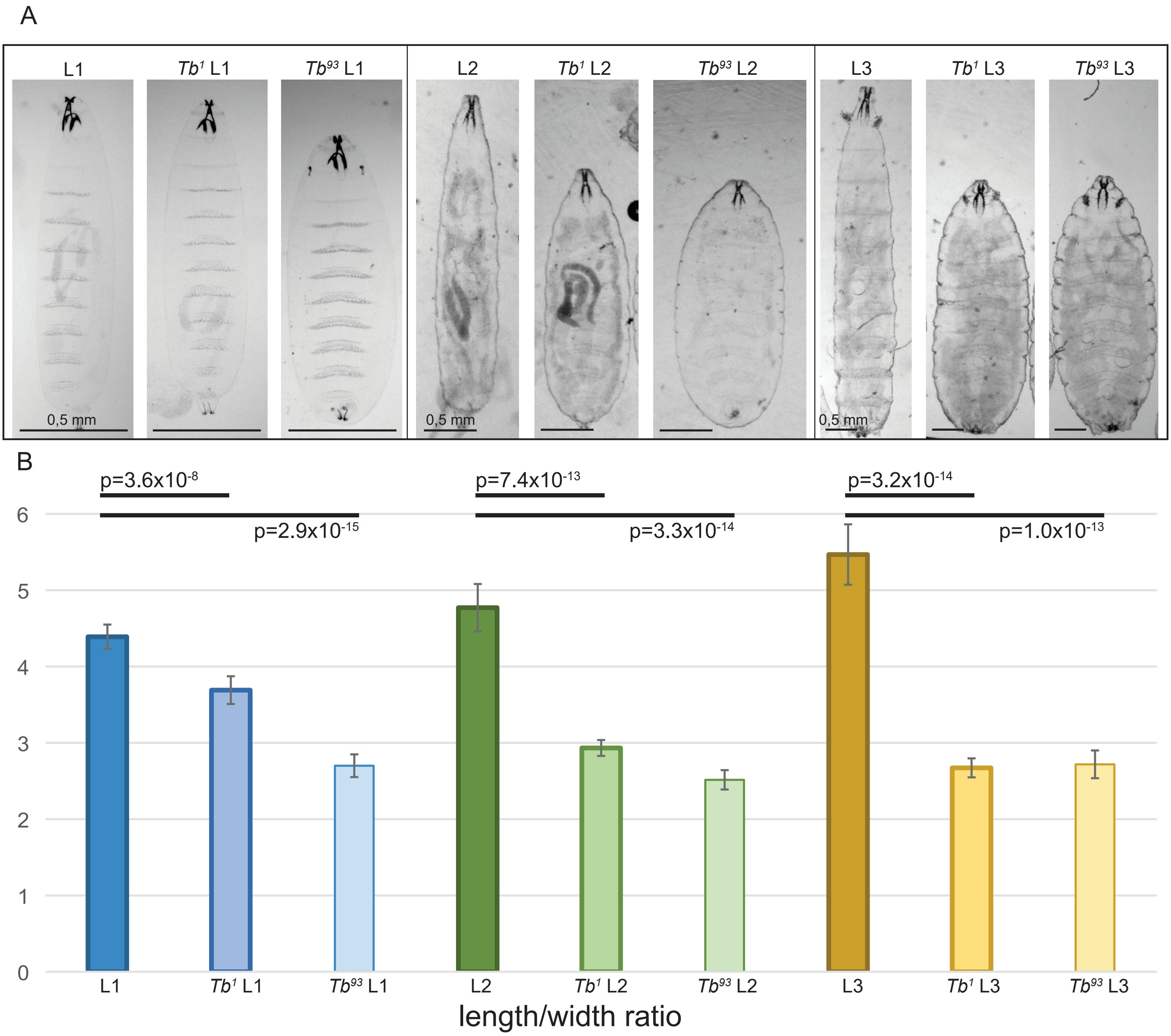
A
L1
Tb^1 L1
Tb^{93} L1
L2
Tb^1 L2
Tb^{93} L2
L3
Tb^1 L3
Tb^{93} L3
0,5 mm
0,5 mm
0,5 mm
B
p=3.6x10-8
p=2.9x10-15
p=7.4x10-13
p=3.3x10-14
p=3.2x10-14
p=1.0x10-13
6
5
4
3
2
1
0
L1
Tb^1 L1
Tb^{93} L1
L2
Tb^1 L2
Tb^{93} L2
L3
Tb^1 L3
Tb^{93} L3
length/width ratio

Suppl. Fig. 2. Tb^{93} allele is a twdlL allele

In a conserved block III of a DUF 243 domain of the TwdlL protein in *Tb^{93}* larvae there is a missense mutation changing tyrosine into cysteine (A, mark on red). This tyrosine is conserved in the Twdl proteins of many insect species (B, conserved tyrosine marked with red frame).

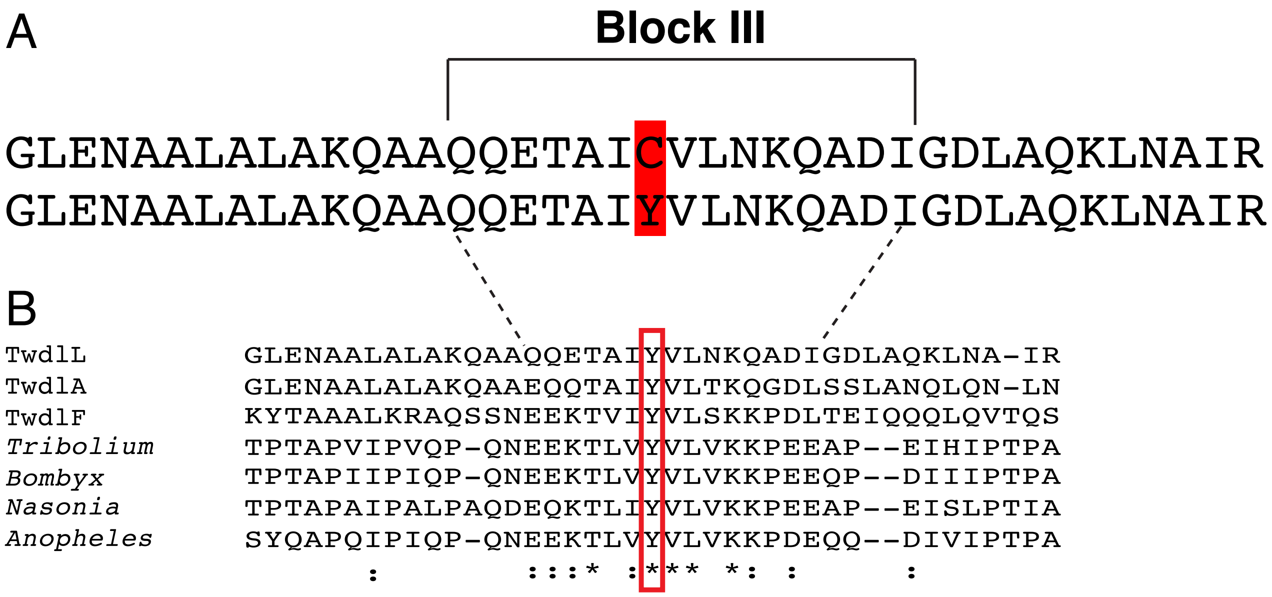
A
Block III
GLENAALALAKQAAQQETAICVLNKQADIGDLAQKLNAIR
GLENAALALAKQAAQQETAIYVLNKQADIGDLAQKLNAIR
B
TwdlL GLENAALALAKQAAQQETAIYVLNKQADIGDLAQKLNA-IR
TwdlA GLENAALALAKQAAEQQTAIYVLTKQGDLSSLANQLQN-LN
TwdlF KYTAAALKRAQSSNEEKTVIYVLSKKPDLTEIQQQLQVTQS
Tribolium TPTAPVIPVQP-QNEEKTLVYVLVKKPEEAP--EIHIPTPA
Bombyx TPTAPIIPIQP-QNEEKTLVYVLVKKPEEQP--DIIIPTPA
Nasonia TPTAPAIPALPAQDEQKTLIYVLVKKPEEAP--EISLPTIA
Anopheles SYQAPQIPIQP-QNEEKTLVYVLVKKPDEQQ--DIVIPTPA
: :::* :*** *: : :

Suppl. Fig. 3. Extended version of Fig. 7 with separated channels

In addition to the images shown in figure 7, this figure shows the single channel images. For more information, please consult the legend of figure 7.

7A

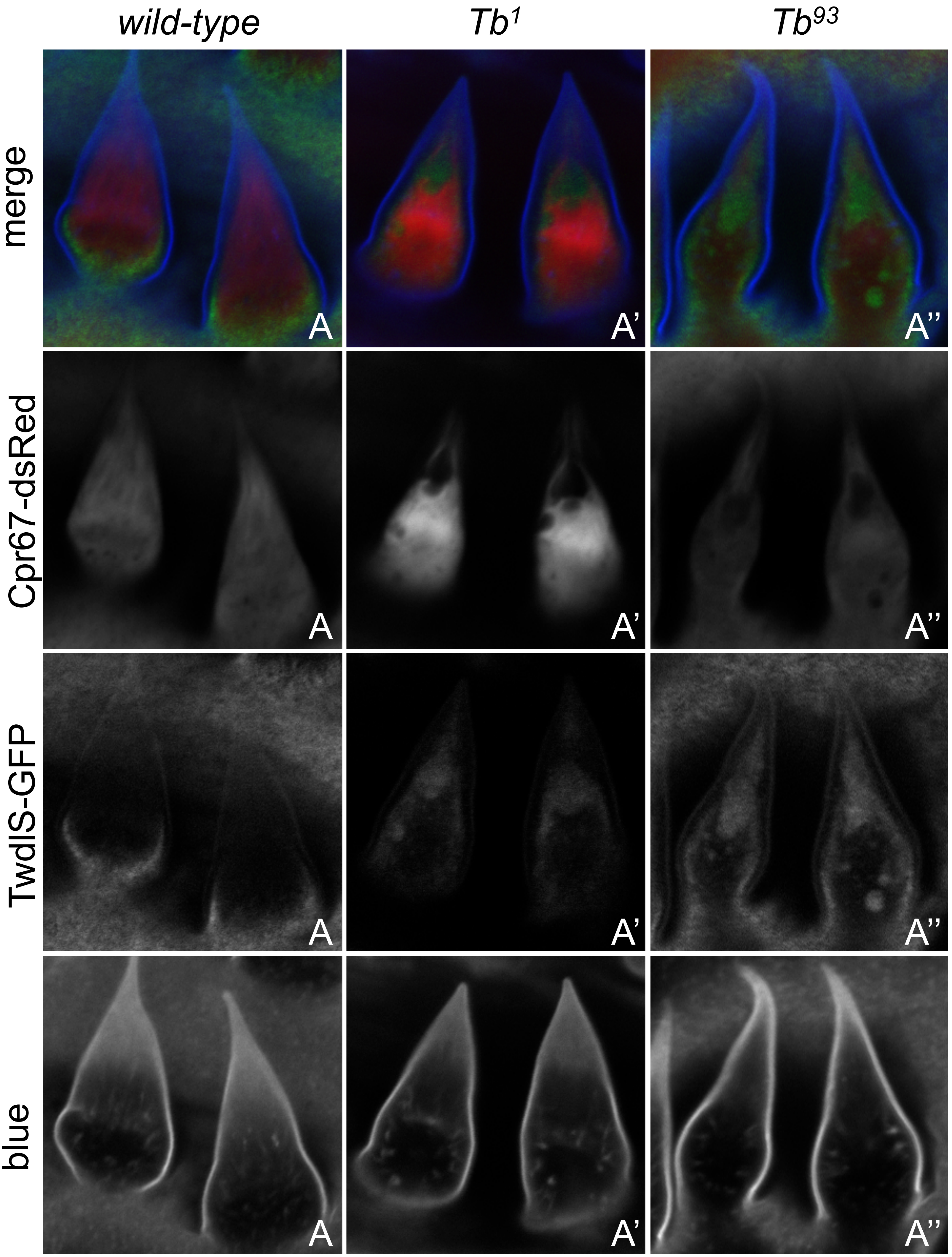

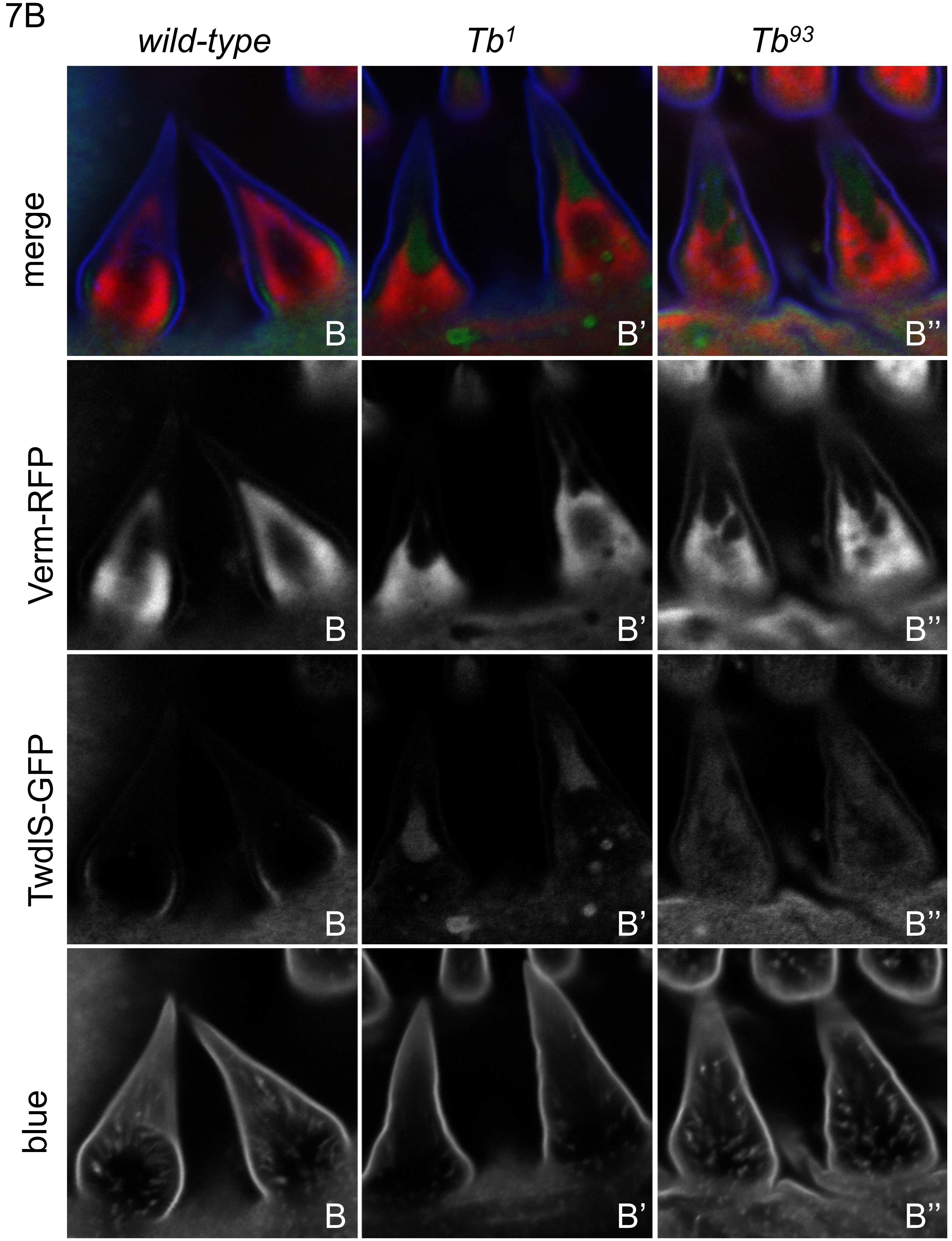
7B
wild-type
Tb1
Tb93
merge
Verm-RFP
TwdlS-GFP
blue
B
B'
B''

7C

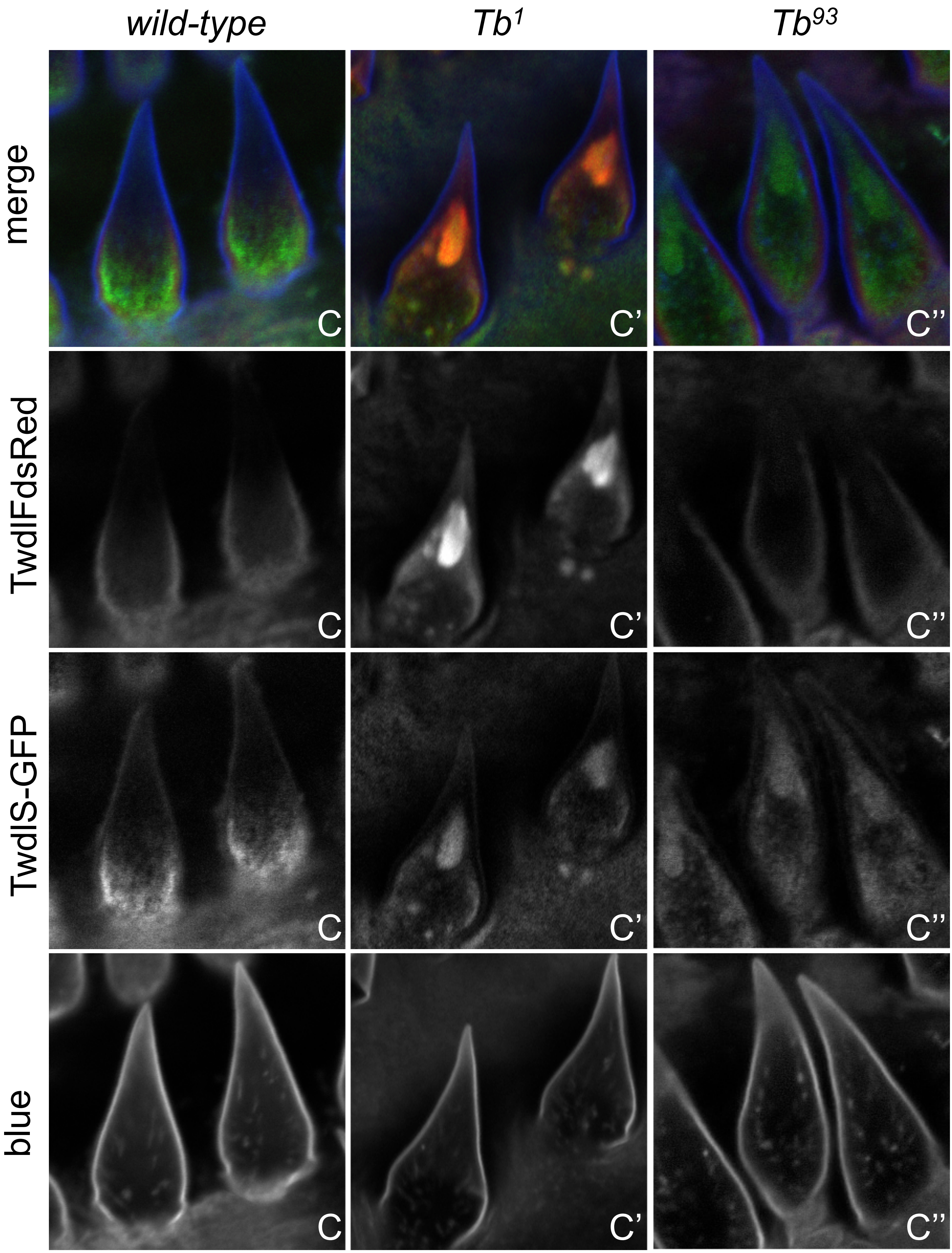

7D

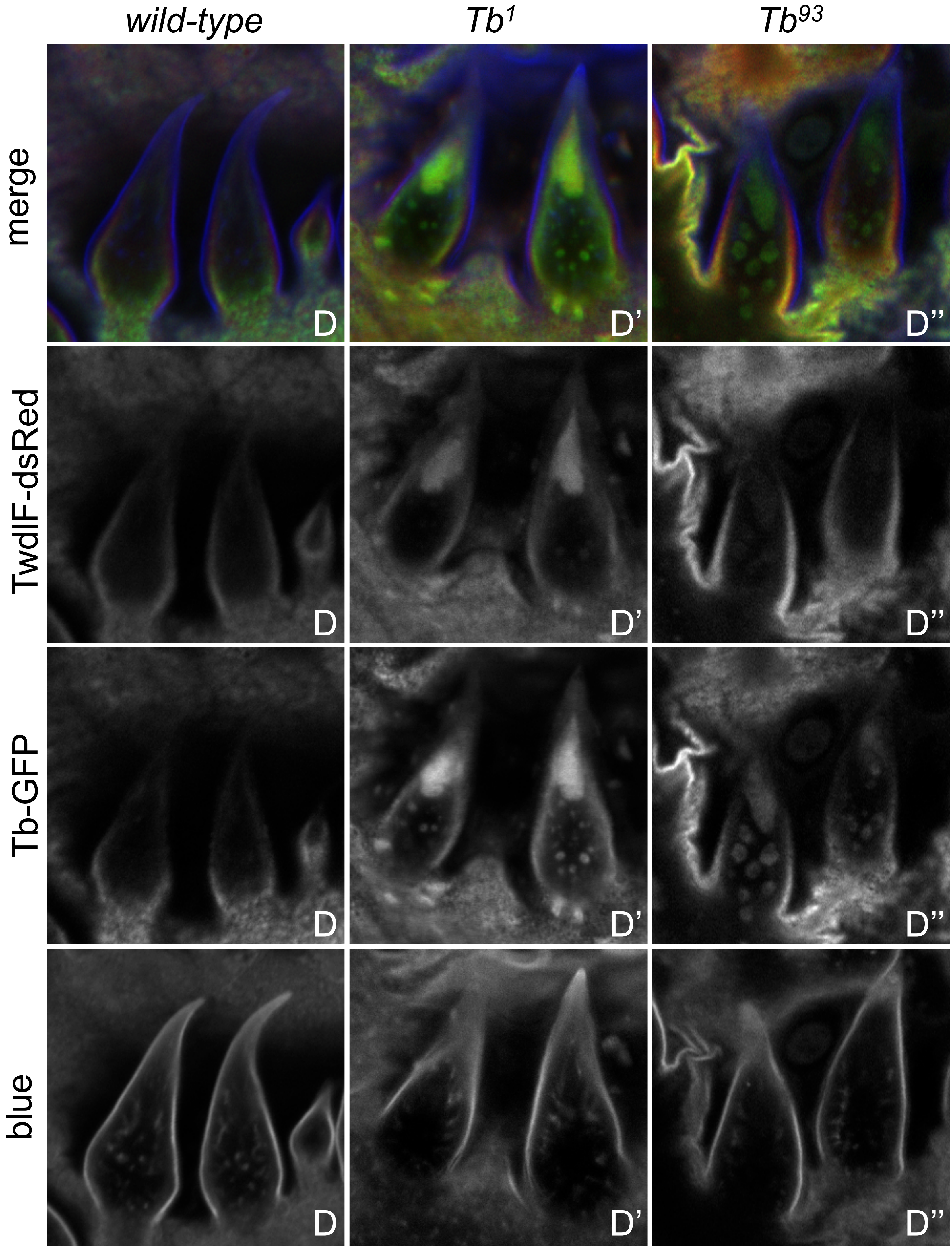

7E & F

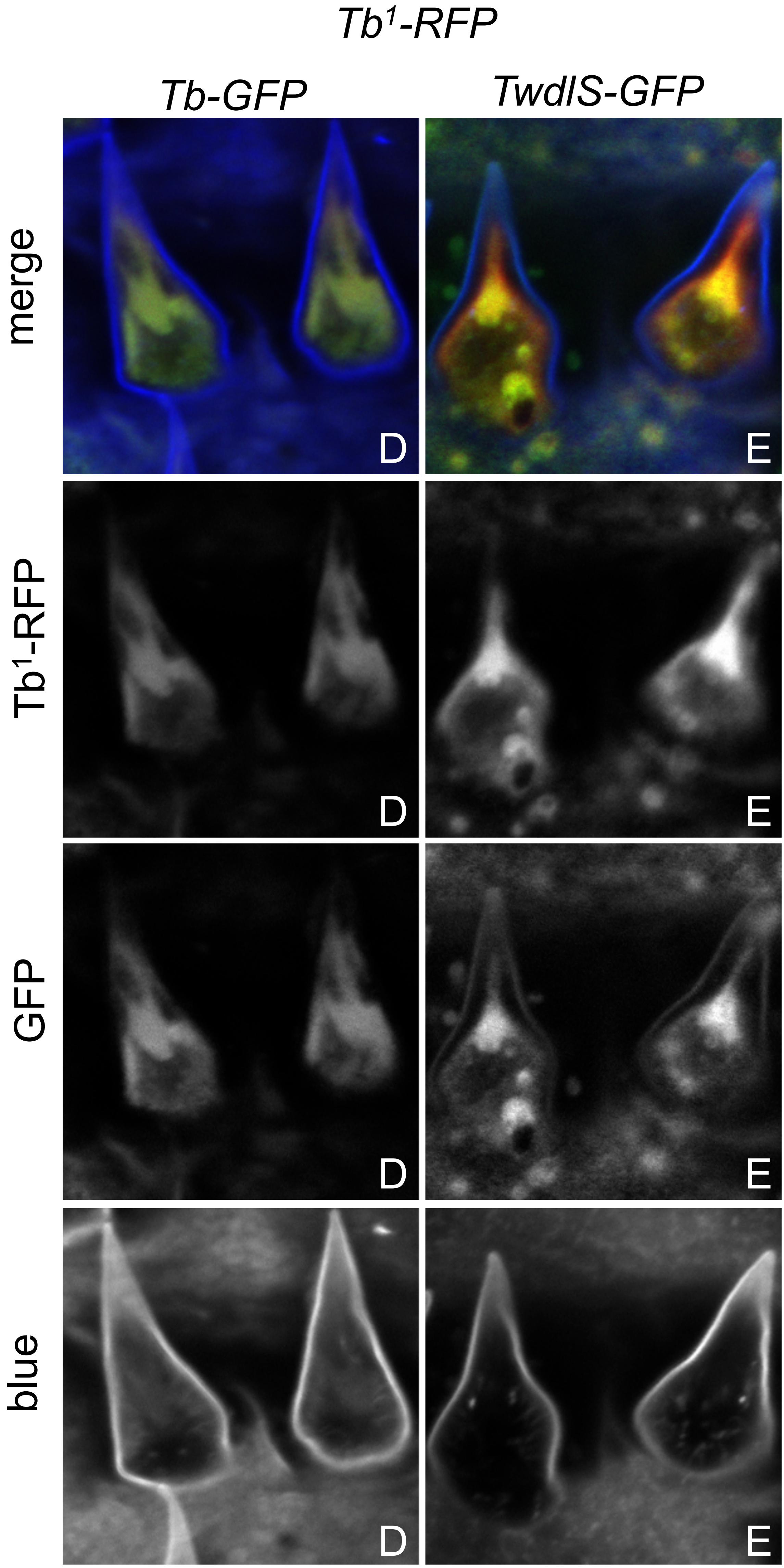

Suppl. Fig. 4. Extended version of Fig. 8 with separated channels

In addition to the images shown in figure 8, this figure shows the single channel images. For more information, please consult the legend of figure 8.

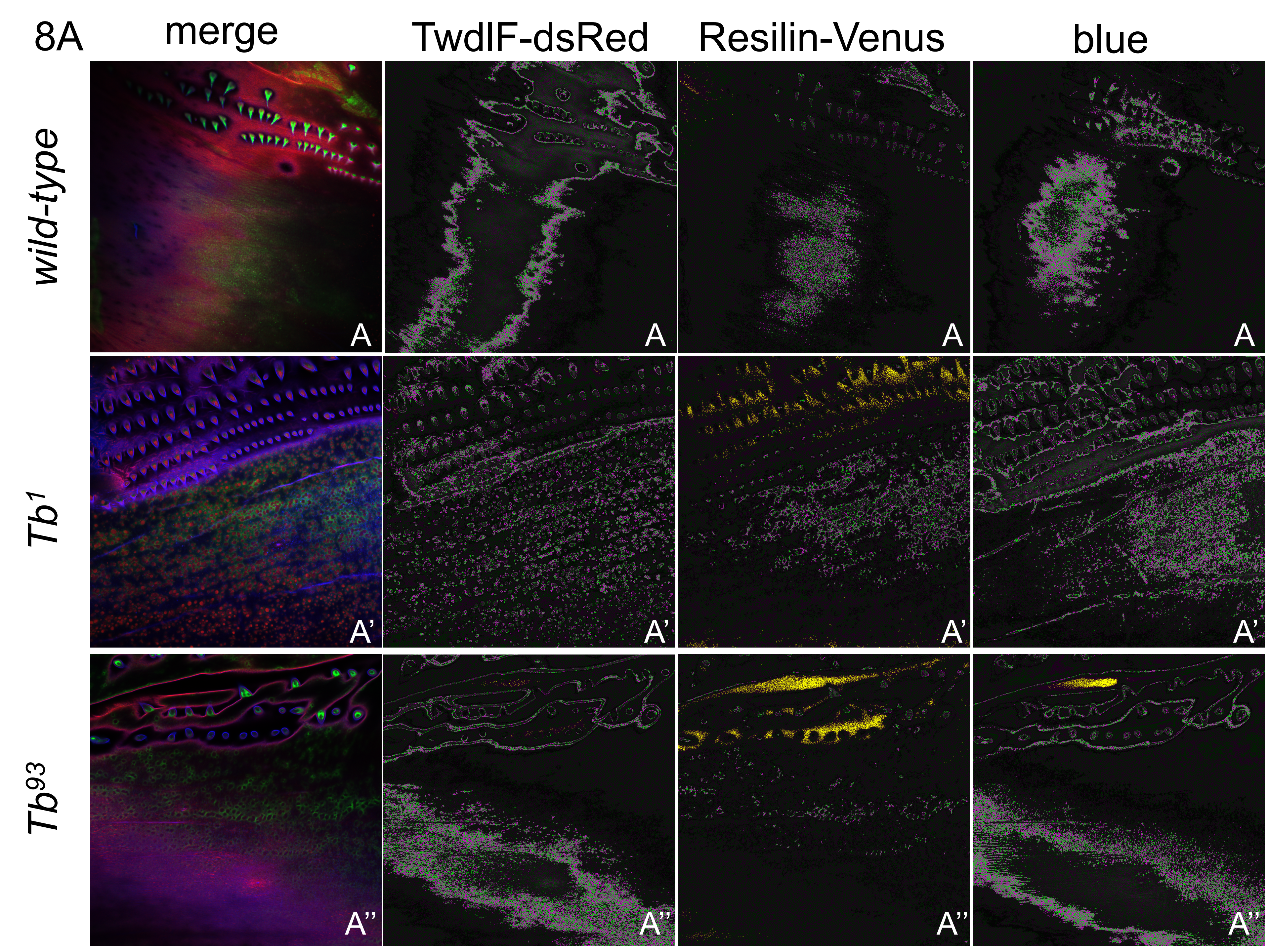

8A
merge
TwdlF-dsRed
Resilin-Venus
blue
wild-type
Tb1
Tb93
A
A'
A''

8B

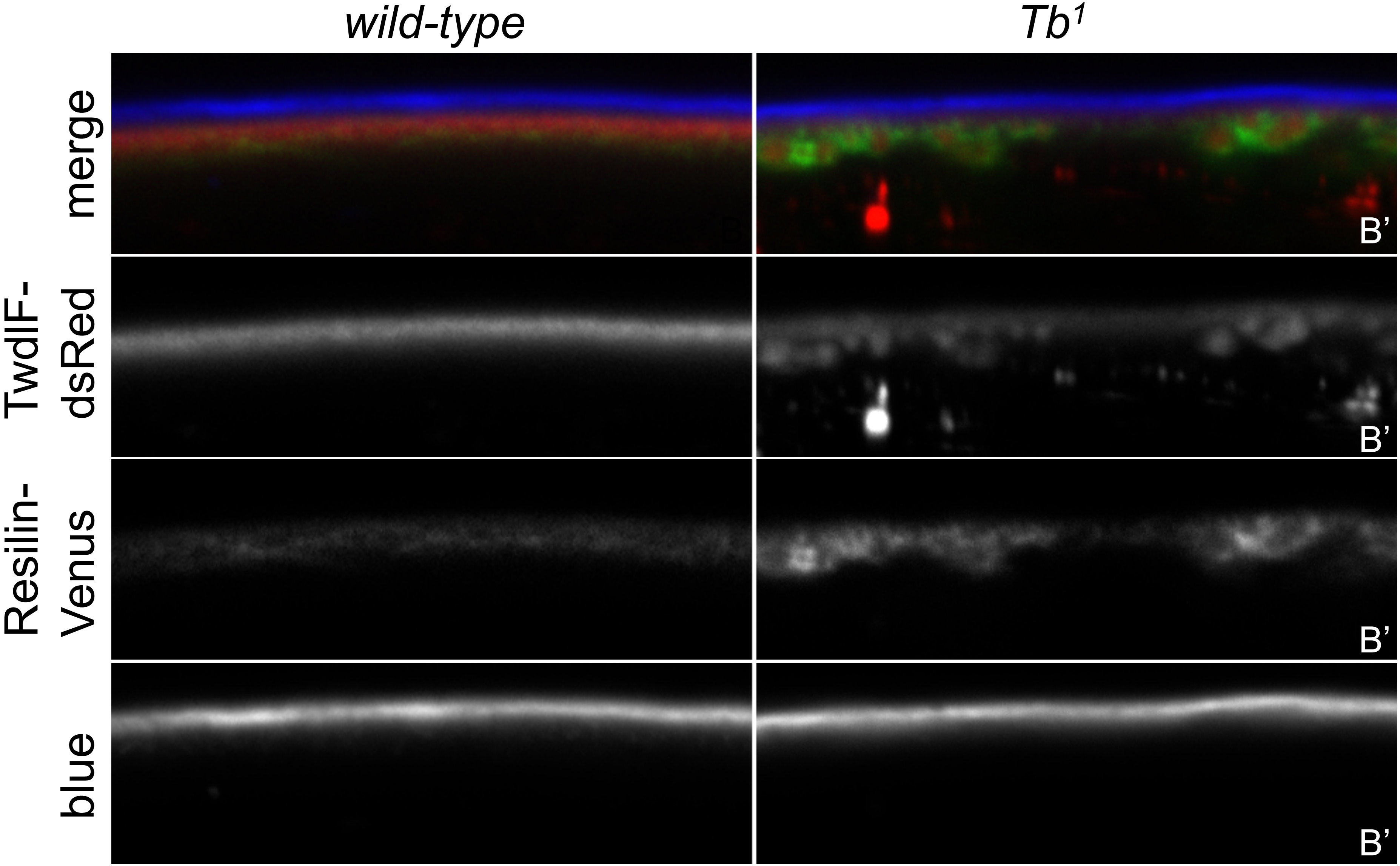

8C

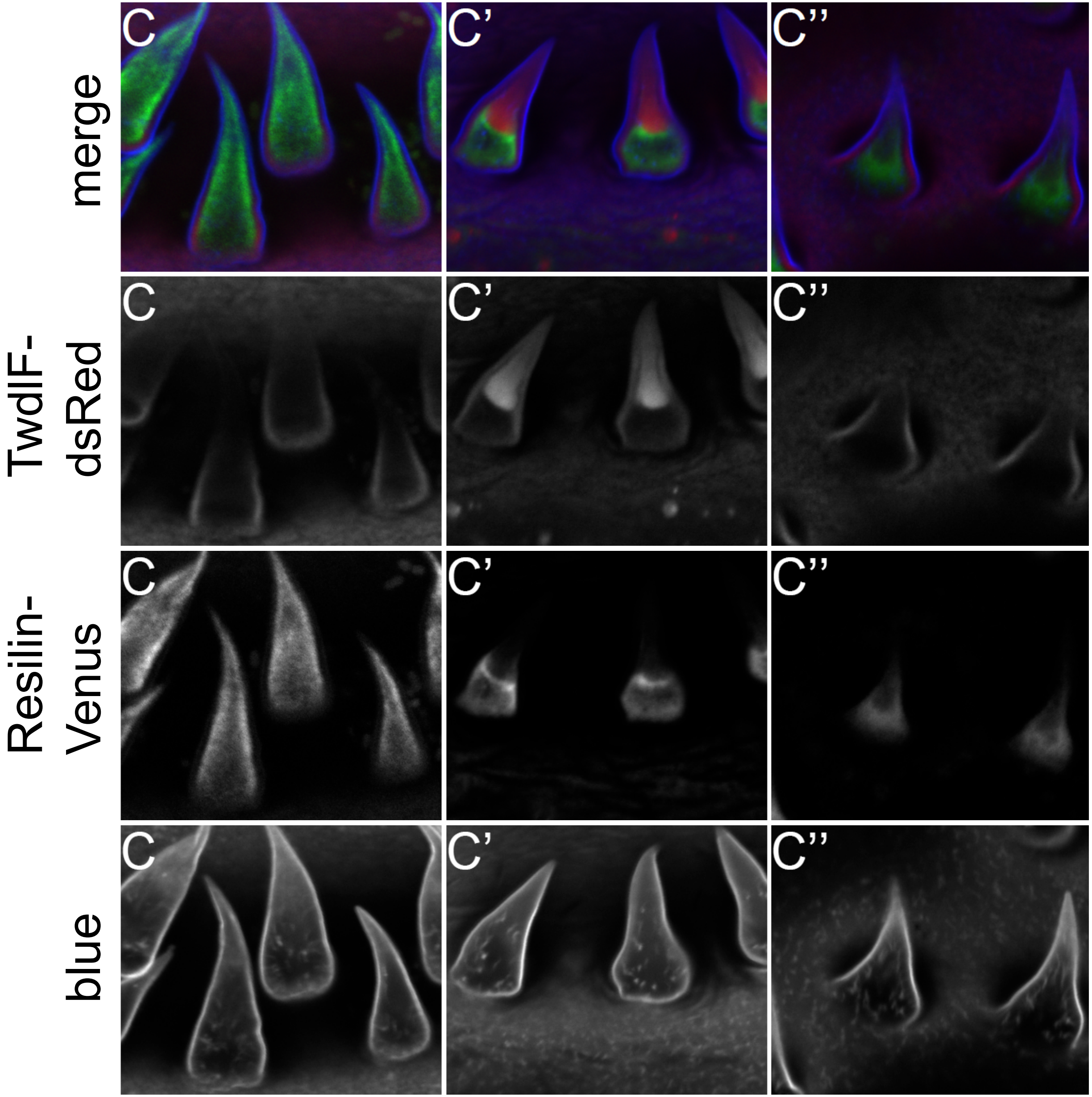

8D

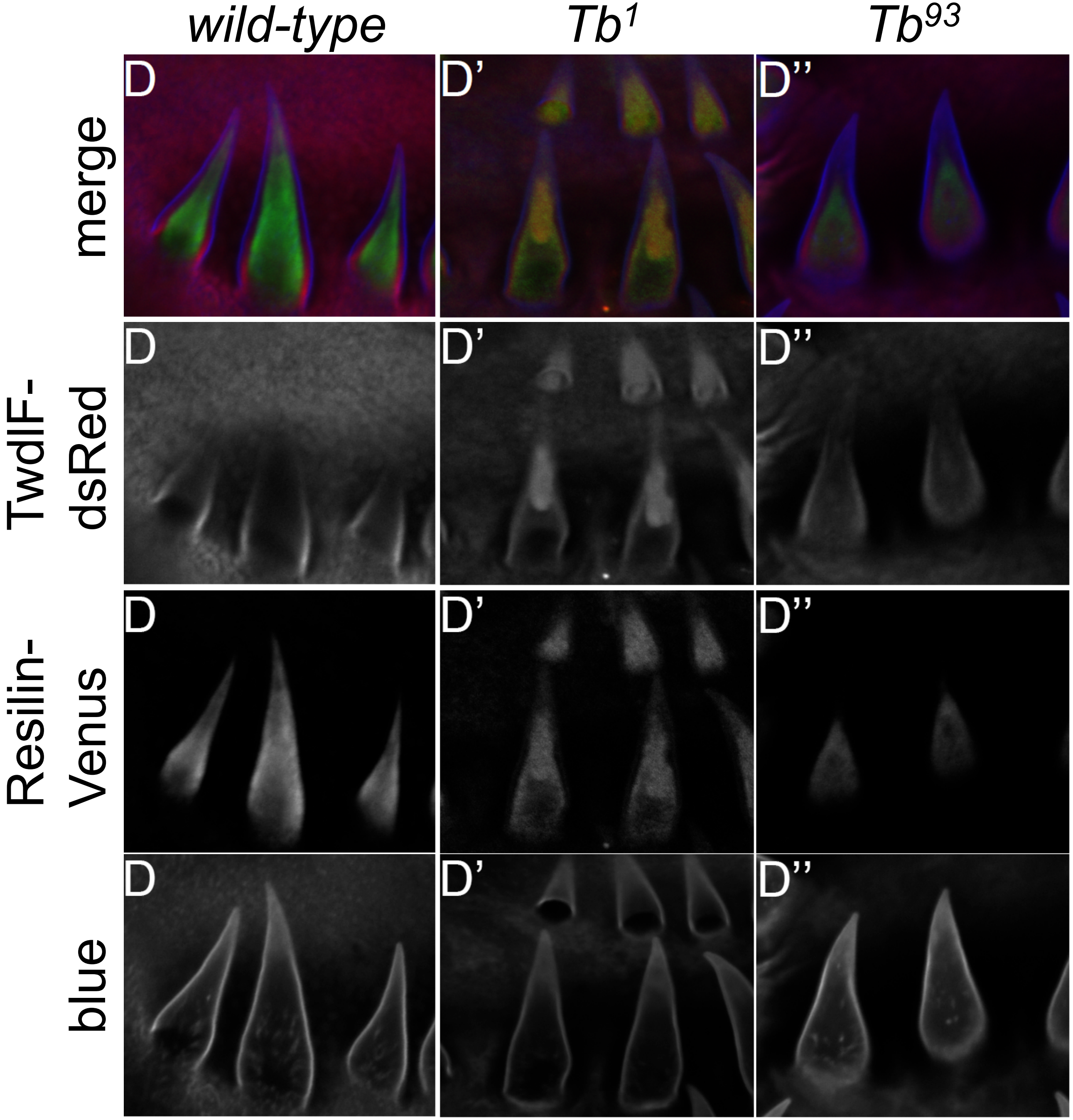

Suppl. Fig. 5. Non-cell autonomous localization of the aggregated Twdls in the cuticle

On the dorsal side of the wild-type L2 larvae TwdlD-dsRed is visible in striped domains of the naked cuticle, excluding the domains with hairs (A; B: magnification of the hair region). In the cuticle of Tb^{1} (A', B') and Tb^{93} (A'', B'') mutant larvae the aggregates of the TwdlD-dsRed are observable in the whole cuticle, also in hairs. Red Fluorescent Protein with attached nuclear localisation signal, expressed from the TwdlD promoter (TwdlD>RFP-NLS) in the background of the wild-type (C), Tb^{1} (C') and Tb^{93} (C'') larvae, in all three cases shows a striped pattern of red nuclei. In control larvae expressing Red Fluorescent Protein with an NLS signal in the whole cuticle, all epidermal red nuclei are visible (C'''; green: ubiquitously expressed membrane-binding CD8-GFP marks the cell borders).

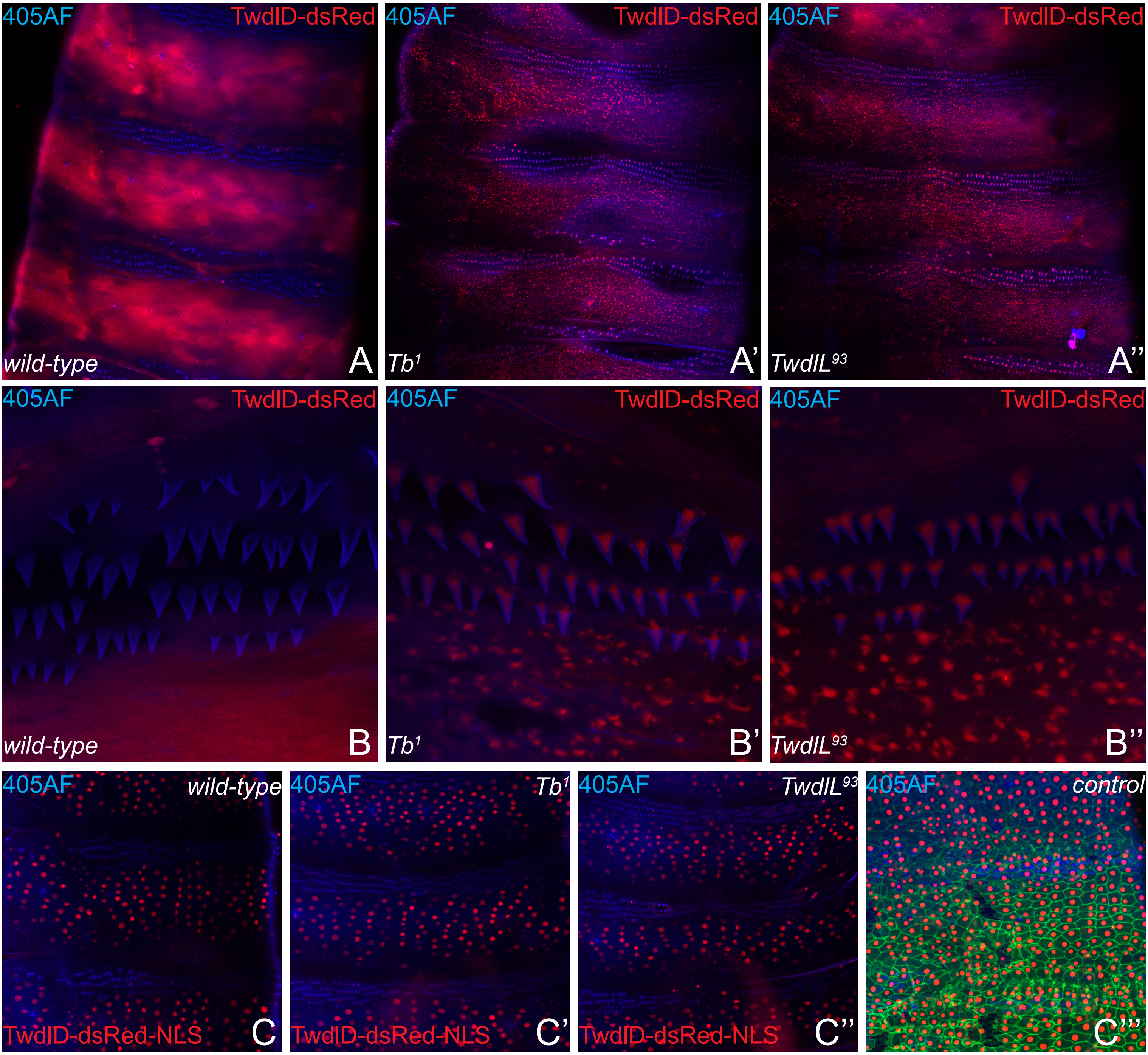

405AF
TwdlD-dsRed
wild-type
A
405AF
TwdlD-dsRed
Tb1
A'
405AF
TwdlD-dsRed
TwdlL93
A''
405AF
TwdlD-dsRed
wild-type
B
405AF
TwdlD-dsRed
Tb1
B'
405AF
TwdlD-dsRed
TwdlL93
B''
405AF
wild-type
TwdlD-dsRed-NLS
C
405AF
Tb1
TwdlD-dsRed-NLS
C'
405AF
TwdlL93
TwdlD-dsRed-NLS
C''
405AF
control
C'''

Suppl. Fig. 6. The differences in the cell shape of wild-type and Tb mutant larvae.

The expression of the mCherry-tagged E-Cadherin (red), accumulating at the borders of epidermal cells shows that the cells of the third instar Tb^{1} (B) and Tb^{93} (C) larvae are flatter longitudinally along the anterior-posterior axis (ant, pos) and broader laterally compared to the wild-type epidermal cells (A). Wild-type epidermal cells marked with CD8-GFP (D) are shorter perpendicularly to the anterior-posterior axis (ant, pos) than epidermal cells of larvae expressing Tb^{1}-RFP (E).

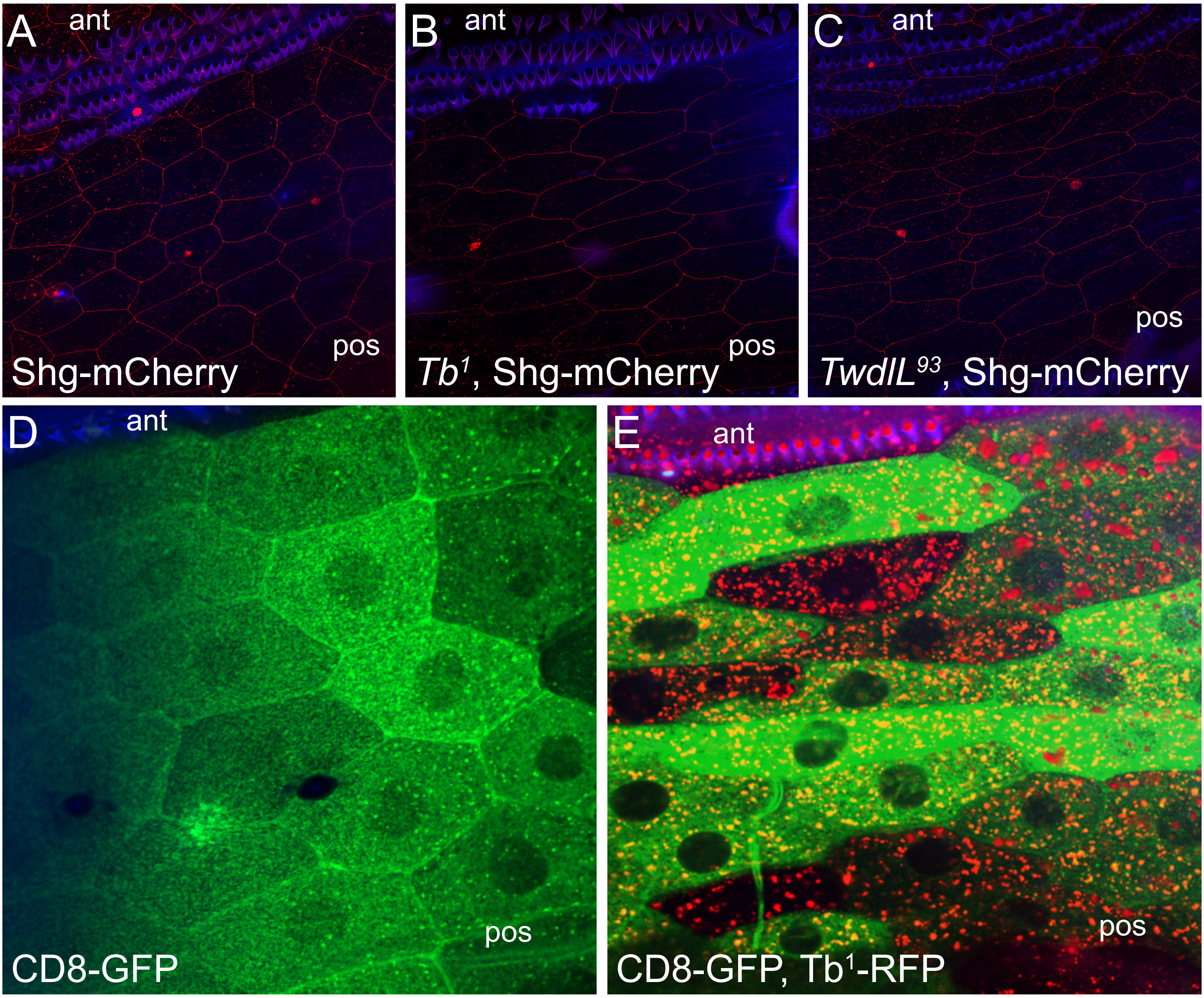
A
ant
Shg-mCherry
pos
B
ant
Tb1, Shg-mCherry
pos
C
ant
TwdlL93, Shg-mCherry
pos
D
ant
CD8-GFP
pos
E
ant
CD8-GFP, Tb1-RFP
pos

Suppl. Fig. 7. Comparison of the cell shape and the cell number in wild-type, Tb^1 and Tb^{93} third instar larvae.

The cell shape and number were determined in the larval third abdominal segment of *Tb^1*, *Tb^{93}* and wild-type larvae (A, B and C, respectively). The ratio of the length to the width of the epidermal cells of *Tb* mutant larvae is significantly lower compared to the ratio of the wild-type epidermal cells (D). The differences are statistically significant following a Student's T-test. The p-values are <0.05 for the comparison between wild-type and *Tb^1* or *Tb^{93}*. The borders of the cells with measured length (along the anterior-posterior axis) and width (perpendicular to the anterior-posterior axis are marked in blue in A-C). The average cell surface area (calculated for the blue marked cells in A-C) also does not differ between the three cases (E). The differences are statistically insignificant following a Student's T-test. The p-values are >0.05 for the comparison between wild-type and *Tb^1* or *Tb^{93}*. The average cell number along the whole segment between the two areas with hairs and across half of the segment is comparable in the epidermis of wild-type, *Tb^1* and *Tb^{93}* mutant larvae (F, G). Borders of counted cells are marked in red in A-C. For both counting experiments, the differences are statistically insignificant following a Student's T-test. The p-values are >0.05 for the comparison between wild-type and *Tb^1* or *Tb^{93}*. Green: ubiquitously expressed membranous CD8-GFP, blue: 405nm-induced cuticle auto-fluorescence.

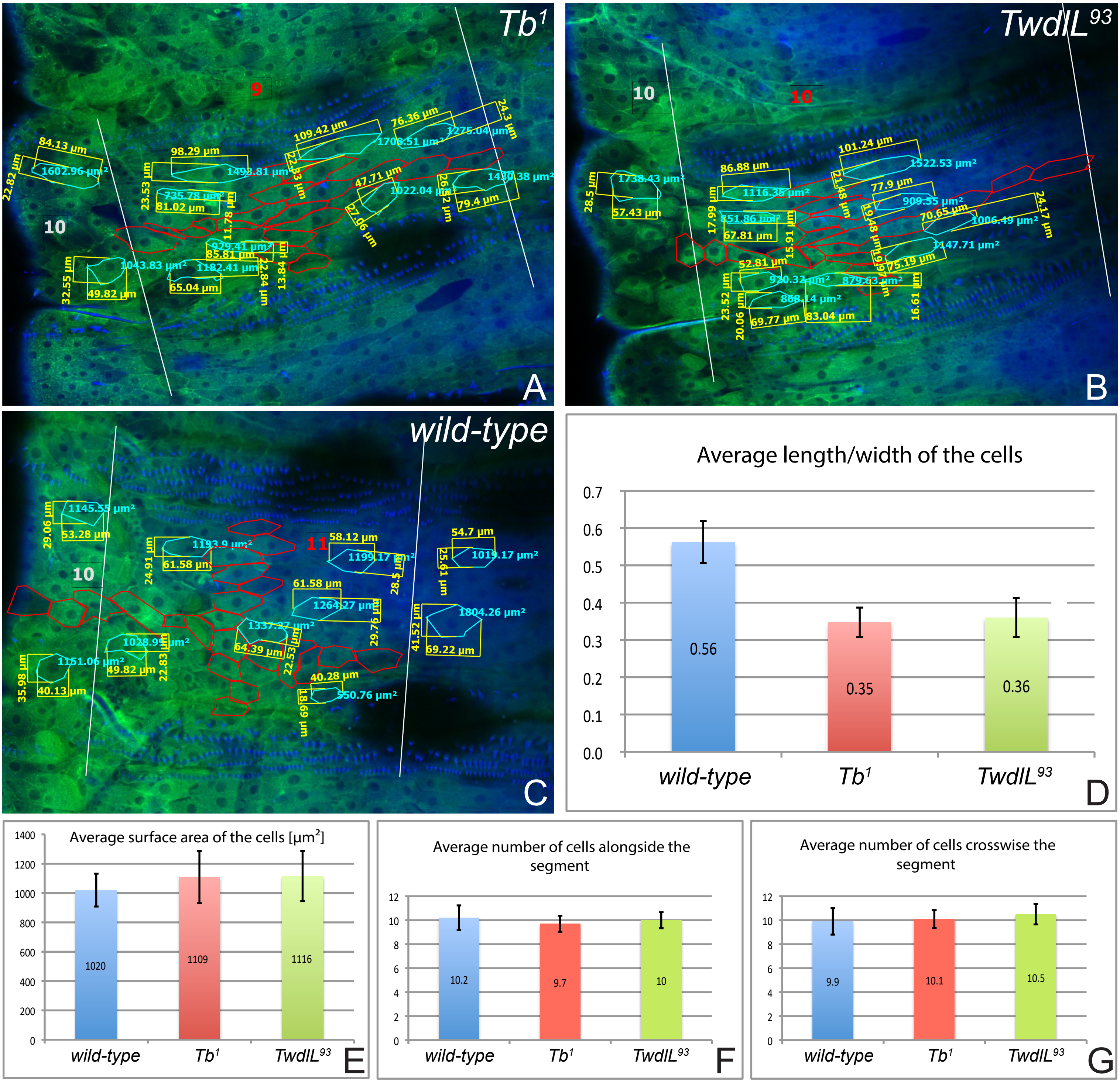

Tb1
A
TwdlL93
B
wild-type
C
Average length/width of the cells
0.7
0.6
0.5
0.4
0.3
0.2
0.1
0.0
0.56
0.35
0.36
wild-type
Tb1
TwdlL93
D
Average surface area of the cells [µm2]
1400
1200
1000
800
600
400
200
0
1020
1109
1116
wild-type
Tb1
TwdlL93
E
Average number of cells alongside the segment
12
10
8
6
4
2
0
10.2
9.7
10
wild-type
Tb1
TwdlL93
F
Average number of cells crosswise the segment
12
10
8
6
4
2
0
9.9
10.1
10.5
wild-type
Tb1
TwdlL93
G

Suppl. Fig. 8. Movement is normal in twdl mutant larvae

(A) During crawling on a substratum, the wild-type larva attains two extrema, one at the maximally contracted and one at the maximally stretched state. The ratio (y-axis: contracted/stretched) of these two extrema is normal in Tb^1 and Tb^{93} mutant larvae (n=5 for each genotype). The differences between the groups are not significant with the p-values of p=0.57 (wild-type versus Tb^1) and p=0.28 (wild-type versus Tb^{93}) after Student's t-test. (B) For pupariation, larvae crawl vertically on the tube wall. On average, wild-type, Tb^1 and Tb^{93} mutant larvae pupariate at the similar distance from the substratum. The differences between the groups are not significant with the p-values of p=0.67 (wild-type versus Tb^1) and p=0.46 (wild-type versus Tb^{93}) after a Student's t-test.

Supplementary figure 4

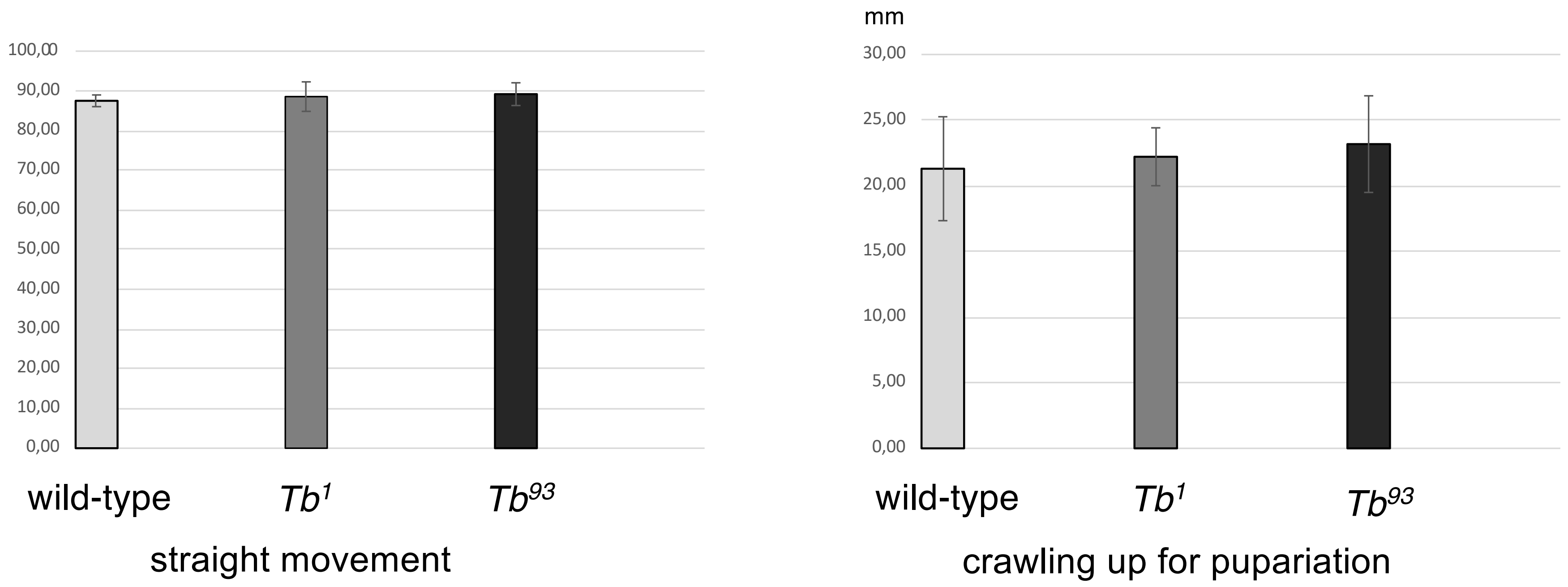

Suppl. Fig. 9. Influence of the ectopic Twdl aggregates on the epidermal apical surface

The apical epidermal surface in wild-type third instar larvae is even (A-A'', green: ubiquitously expressed membranous CD8-GFP marking the apical cell surface, blue: 405nm-induced auto-fluorescence of the envelope; A': without red channel; A'': without green channel). Tb^1-RFP aggregates (red) accumulating ectopically in the procuticle cause the convexities in the epidermal cell surface (B-B''; without red (B') and green channel (B'')); convexity shown by a white arrow). C-C'': the top view, convexities in the epidermal apical surface shown by a white arrow, whilst the cellular Tb^1-RFP aggregates shown by a yellow arrow. D-D''': Z-stack of the epidermal cells from the top view with higher magnification, showing procuticular aggregates and the cellular aggregates inside the cell. E-E''': Z-stack without a red channel, revealing notches in the epidermal surface in the places of the procuticular aggregates.

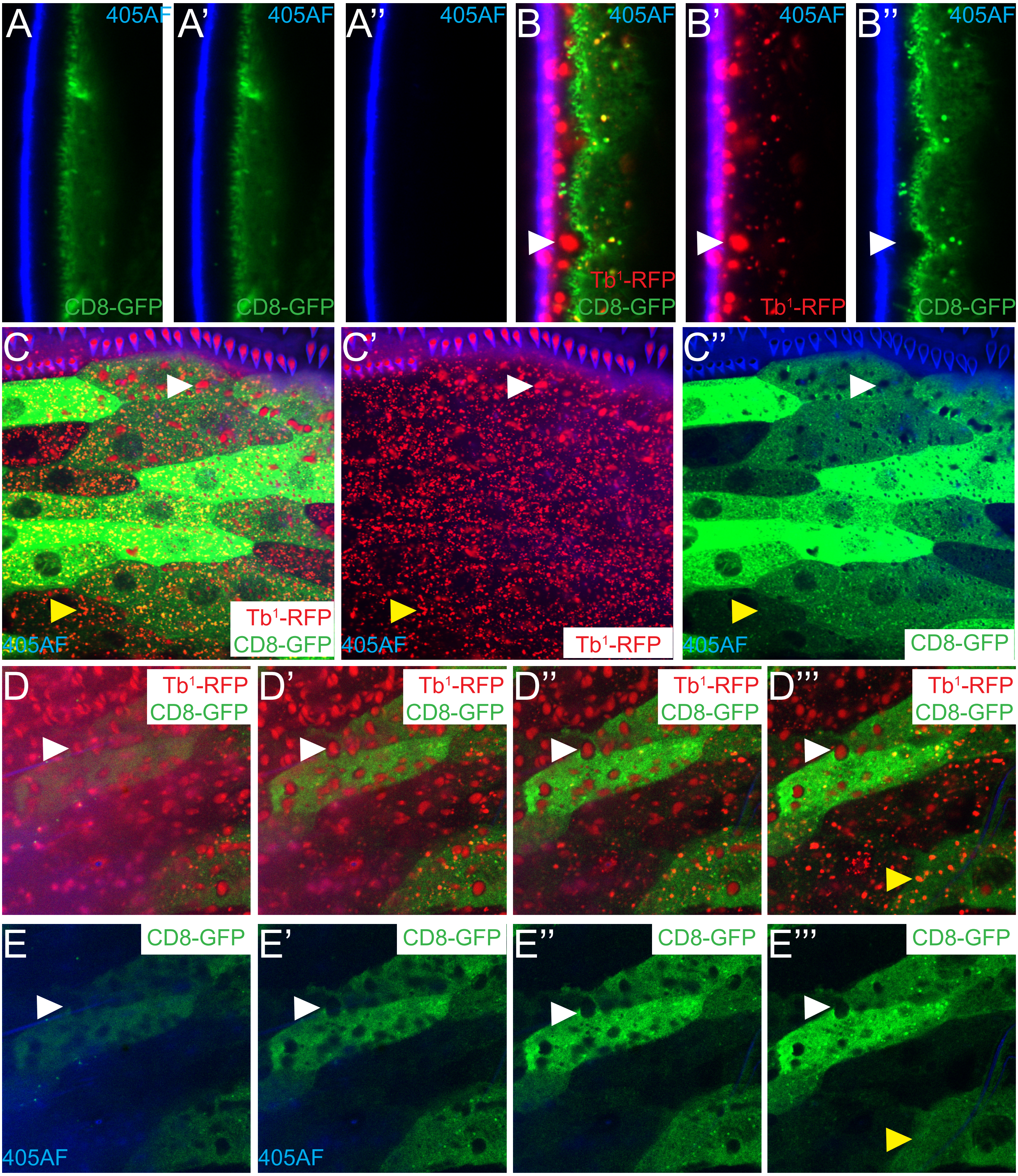
A
405AF
CD8-GFP
A'
A''
B
B'
B''
Tb1-RFP
C
C'
C''
D
D'
D''
D'''
E
E'
E''
E'''

Suppl. Fig. 10. The twdl genomic region on chromosome 3.

The *twdl* gene cluster on the right arm of chromosome 3 encompasses 14 *twdl* genes including *Tb* (*twdlA*). In this work, we identified *twdlL* (red box) as the gene mutated in Tb^{93}. Two Flyfos constructs with genomic DNA flanking *gfp*-tagged versions of *twdlS* (light grey regions 23.34 kb 5' and 16.52 kb 3' of the gene, green box) and *Tb* (dark grey regions 40.18 kb 5' and 11.86 kb 3' of the gene, green box) were used for Twdl localisation experiments in live larvae.

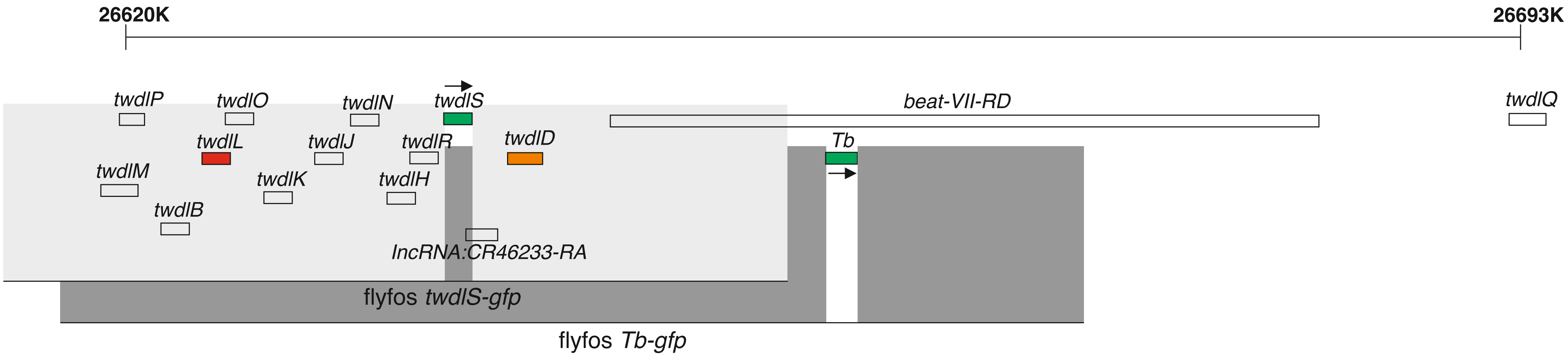
26620K
26693K
twdlP
twdlO
twdlN
twdlS
beat-VII-RD
twdlQ
twdlL
twdlJ
twdlR
twdlD
Tb
twdlM
twdlK
twdlH
twdlB
lncRNA:CR46233-RA
flyfos twdlS-gfp
flyfos Tb-gfp

Suppl. Table 1. Primer sequences for amplification and sequencing of twdl genes.

To identify a possible mutation in a *twdl* gene in *Tb*^{93} flies, DNA fragments of *twdl* genes in the *twdl* cluster on chromosome 3 were amplified by standard PCR with wild-type and *Tb*^{93} genomic DNA as templates. Amplicons were sequenced by Macrogen (South Korea).

Primer	Sequence
TwdlQleft	GAAGATGAAACGGAAACCGA
TwdlQright	TGAAACCTCTTCTCGTTCCG
TwdlDleft	GTCTATTTCGTGGGAGGGGT
TwdlDright	AAGAGAAACTCGCACAACGG
TwdlSleft	AGCAGGCAAACAAACACACA
TwdlSright	GCGAAATCAAAAGGCTAACC
TwdlRleft	AGTTGGTTGGGAATAGCGAA
TwdlRright	GCCGCTTACCGAAATTAACA
TwdlHleft	CGAAAGAAGCCAACTCCAAA
TwdlHright	TTTTGCCCTCAAATGTGGTT
TwdlNleft	AAACAGGTTAGGCTCGCAGA
TwdlNright	ACTTGTATTCCATTTGGCGC
TwdlJleft	CGCTTTATGCAGAGCGTTTT
TwdlJright	GCACAAGAATGTCGGGAATT
TwdlKleft	GCAGCAGTGTGACGAATCAT
TwdlKright	TGTTTGGATTTGTTTCGGCT
TwdlOleft	GACTGGATGTATTGGCGAGAA
TwdlOright	CAGAGCAATTCAGCTTTGCA
TwdlLleft	TGCAAAGCTGAATTGCTCTG
TwdlLright	CCACTCATGTTGTCATCGGA
TwdlBleft	TGGCTTCAAAGAAATCTCCG
TwdlBright	ACAAGGCGGACTTAATGCAC
TwdlPleft	TAACAGCATTCCGAAAACCC
TwdlPright	ACCAGTTTATGGCTGCTGAGA
TwdlMleft	AGCTGCCGAAGAAGTTACCA
TwdlMright	CAATGAACTCAGCCTGCAAA
